# High-capacity and reusable nickel borate adsorbent for sulfonated anionic dye removal: comparative adsorption, kinetic, thermodynamic, and regeneration studies

**DOI:** 10.1039/d6ra07924e

**Published:** 2026-09-28

**Authors:** Ayhan Kocaman, Ertuğrul Esmeray, Hakan Çelebi, Mustafa Karataş, İlhan Pekgözlü

**Affiliations:** a Department of Environmental Engineering, Faculty of Engineering and Natural Sciences, Karabük University 78050 Karabük Türkiye ayhankocaman@karabuk.edu.tr ilhanpekgozlu@karabuk.edu.tr; b Department of Civil Engineering Faculty of Engineering and Natural Sciences, Karabük University 78050 Karabük Türkiye eesmeray@karabuk.edu.tr; c Department of Environmental Engineering, Faculty of Engineering, Aksaray University 68100 Aksaray Türkiye hakancelebi@aksaray.edu.tr mustafakaratas@aksaray.edu.tr

## Abstract

The study conducted a comparative investigation into the performance of a newly developed nickel borate (Sr_2_Ni(BO_3_)_2_, NiB) adsorbent for the removal of two anionic dyes, Trypan Blue and Ponceau S, from aqueous solutions. The adsorption experiments assessed various parameters, including contact time, pH, adsorbent dosage, agitation speed, temperature, initial dye concentration, and regeneration cycles. High removal efficiencies for both dyes were typically observed under conditions approaching neutral pH, at an agitation speed of 250 rpm and a temperature of 25 °C. In contact-time experiments, the removal efficiencies reached 93.72% for Trypan Blue and 90.54% for Ponceau S within 60 min. Regarding pH studies, Trypan Blue removal was 91.55% at pH 7.50, while Ponceau S removal was 89.72%. Isotherm calculations, adjusted for actual initial concentrations, indicated that at an initial dye concentration of 100 mg L^−1^, the highest experimental adsorption capacities were 463.08 mg g^−1^ for Trypan Blue and 463.85 mg g^−1^ for Ponceau S, with removal rates exceeding 92% for both dyes. The equilibrium data were further evaluated using the non-linear forms of the Langmuir, Freundlich, Temkin, Dubinin–Radushkevich, Sips, and Redlich–Peterson isotherm models. Because a clear saturation plateau was not reached within the tested concentration range, model-derived maximum capacity parameters were interpreted cautiously as apparent estimates rather than definitive maximum adsorption capacities. In the kinetic evaluation, the adsorption data were assessed using non-linear kinetic fitting and error-function-based model validation. The results indicated that the adsorption process was better interpreted as a multi-step mechanism involving rapid surface attachment, surface rearrangement, and diffusion-related contributions rather than as a single rate-controlling process. Thermodynamic analysis confirmed the spontaneous, exothermic nature of the adsorption process. In regeneration experiments, thermal treatment outperformed HCl regeneration, maintaining removal efficiencies of 72.5% for Trypan Blue and 68.0% for Ponceau S by the ninth cycle. Overall, these findings demonstrate that NiB is a high-capacity, multi-mechanism, and reusable inorganic adsorbent candidate for the removal of sulfonated anionic dyes.

## Abstract 2

The study conducted a comparative investigation into the performance of a newly developed nickel borate (Sr_2_Ni(BO_3_)_2_, NiB) adsorbent for the removal of two anionic dyes, Trypan Blue and Ponceau S, from aqueous solutions.

## Graphical abstract

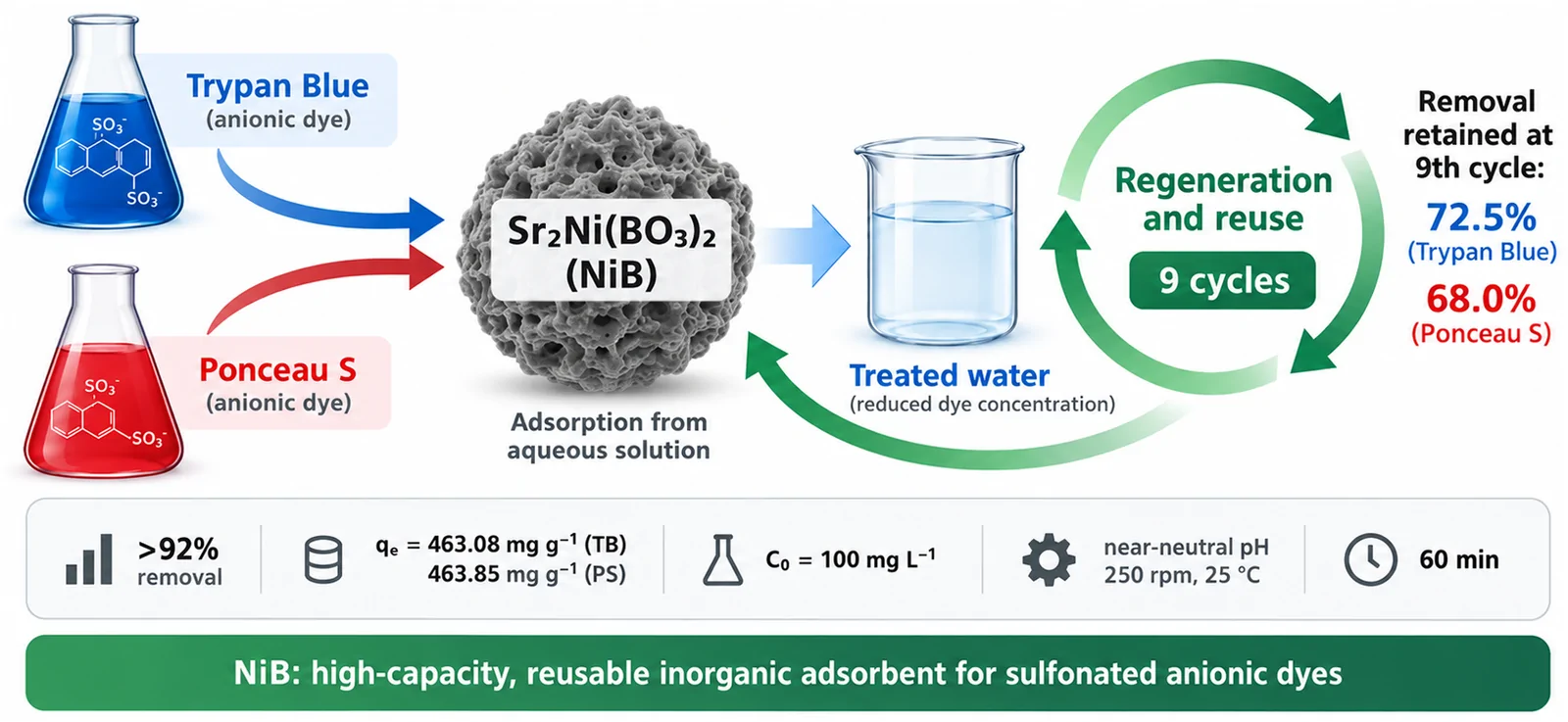

## 1. Introduction

Wastewater generated from textile, paint, paper, food, and cosmetic industries often contains synthetic dyes with high color intensity, complex aromatic structures, and resistance to conventional treatment. Even at low concentrations, these dyes reduce light penetration in aquatic systems, disrupt photosynthetic activity, affect the balance of dissolved oxygen, and may generate toxic, mutagenic, or persistent transformation products.^1,2^ Several treatment methods, including coagulation–flocculation, membrane filtration, biological treatment, advanced oxidation, electrochemical processes, and adsorption, have been applied for dye removal. However, many of these techniques are limited by high chemical or energy demand, sludge production, membrane fouling, catalyst instability, or limited reusability.^3^

Adsorption remains one of the most practical approaches for treating dye-contaminated water due to its operational simplicity, high removal efficiency, and compatibility with various dye classes. Adsorption performance is governed by the physicochemical properties of the adsorbent, including surface area, pore structure, surface functional groups, surface charge, crystallinity, and chemical stability, as well as by operating conditions such as solution pH, initial dye concentration, adsorbent dosage, contact time, stirring speed, and temperature.^4^ Therefore, current adsorption studies increasingly focus not only on removal efficiency but also on kinetic behavior, equilibrium modeling, thermodynamic feasibility, regeneration capacity, and adsorption mechanisms. Recent adsorption-based studies have further emphasized that adsorbent performance should be evaluated together with material sustainability, regeneration/reuse behavior, post-use management, and scale-up potential, including fixed-bed or continuous-flow operation, rather than relying only on batch removal efficiency.^5,6^

Low-cost biopolymer-based adsorbents, waste-derived activated carbons, magnetic composites, metal oxides, ferrites, and hybrid inorganic materials have been investigated for the removal of anionic and cationic dyes.^7–12^ Recent advances in nanotechnology-assisted and inorganic adsorbent systems have also shown that tunable surface chemistry, high reactive-site density, structural stability, and functional surface modification are important criteria for improving contaminant removal in water and wastewater treatment applications.^13^ Despite these developments, the design of chemically stable and reusable inorganic adsorbents with well-defined surface interactions remains important for practical dye treatment. Accordingly, mineral-derived and oxide-modified adsorbents have recently been evaluated not only under batch conditions but also in relation to adsorption kinetics, equilibrium behavior, reusability, and process scalability.^6,14^ Trypan Blue and Ponceau S are suitable model pollutants for evaluating anionic dye adsorption because both contain aromatic structures and sulfonate groups but differ in molecular size, spatial configuration, and possible surface orientation. Trypan Blue is a high-molecular-weight anionic azo dye with persistent coloration in aqueous media, whereas Ponceau S is a highly soluble sulfonated azo dye that can interact with adsorbent surfaces through electrostatic attraction, hydrogen bonding, Lewis acid–base interactions, and surface complexation.

Previous studies have reported several routes for Trypan Blue removal, including biological decolorization, biosorption, protein-based sorbents, photocatalytic systems, and hybrid treatment processes.^15,16^ Similarly, the adsorption of Ponceau S and related sulfonated azo dyes has been associated with adsorbent surface chemistry, pH-dependent surface charge, functional group distribution, and mass transfer behavior.^17–19^ These studies confirm that anionic azo dye removal is controlled by multiple interactions rather than by a single adsorption pathway. However, comparative adsorption studies using borate-based inorganic adsorbents for Trypan Blue and Ponceau S remain limited.

Borate-based inorganic materials are structurally versatile solids in which B–O networks can combine with different metal centers to form chemically and thermally stable crystalline phases. Recent studies have shown that metal borate materials, including Sr_2_Mg(BO_3_)_2_, can adsorb anionic dyes such as Reactive Red 195 and Reactive Yellow 145, indicating their potential use as functional adsorbents beyond their conventional structural or optical applications.^20–22^ Nevertheless, the adsorption behavior of nickel borate toward sulfonated anionic dyes remains insufficiently clarified. Nickel borate may provide several interaction sites for dye retention, including Lewis-acidic Ni centers, B–O groups, surface hydroxyls, and heterogeneous surface domains. These sites may contribute to electrostatic attraction, hydrogen bonding, surface complexation, Lewis acid–base interactions, and intraparticle diffusion, particularly under pH-dependent protonation/deprotonation conditions.

The study evaluates a newly developed nickel borate adsorbent for the removal of Trypan Blue and Ponceau S from aqueous solutions. The effects of contact time, solution pH, adsorbent dosage, stirring speed, temperature, and initial dye concentration were investigated to compare the adsorption behavior of the two anionic dyes. Kinetic, equilibrium, thermodynamic, regeneration, and FTIR-based analyses were used to interpret adsorption performance and possible interaction mechanisms. In addition, both non-linear kinetic and isotherm models were compared using multiple error functions to reduce model-selection bias and to provide a more reliable interpretation of adsorption behavior. This integrated evaluation is consistent with recent adsorption studies in which kinetic modeling, equilibrium analysis, thermodynamic assessment, and regeneration performance are considered together to support adsorbent applicability.^6,14^ The study addresses a specific gap in the literature by comparing two sulfonated anionic dyes on the same NiB adsorbent and by linking removal efficiency with adsorption modeling, regeneration behavior, and mechanistic interpretation. This approach provides a clearer assessment of the applicability of NiB as an inorganic adsorbent for the removal of a resistant anionic dye.

## 2. Materials and methods

A schematic overview of the experimental workflow adopted in this study is presented in Scheme 1.

**Scheme 1:**
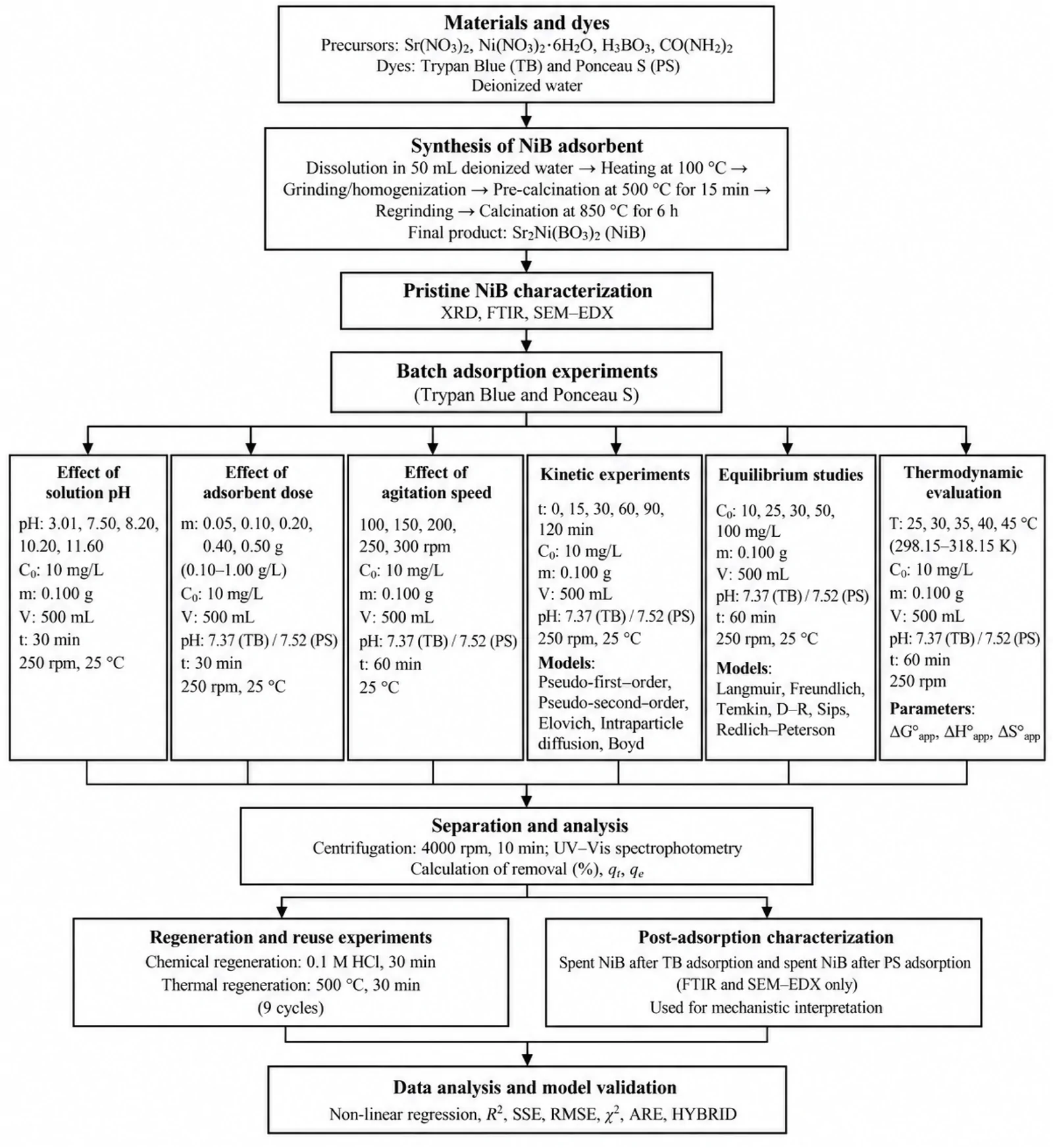
Experimental workflow for NiB-mediated adsorption of Trypan Blue and Ponceau S.

### 2.1. Chemicals and preparation of dye solutions

Trypan Blue and Ponceau S were used as model sulfonated anionic dyes to evaluate the adsorption performance of the synthesized NiB adsorbent.Both dyes were supplied by Merck KGaA (Darmstadt, Germany). Both dyes were selected because they contain aromatic structures and anionic sulfonate groups but differ in molecular size, sulfonate group distribution, and possible surface orientation during adsorption. Ultrapure water produced using a Millipore Elix Advantage 5-Synergy UV system was used for the preparation of all dye solutions. Stock dye solutions were prepared from analytically pure commercial dyes and diluted to the required concentrations before each experiment. The initial dye concentration used in the main adsorption experiments was 10 mg L^−1^. For the equilibrium adsorption experiments, working solutions were prepared at initial concentrations of 10, 25, 30, 50, and 100 mg L^−1^. The pH of the solutions was adjusted using 0.1 M NaOH prepared from analytical-grade NaOH (98%, Merck KGaA, Darmstadt, Germany) and 0.1 M HCl prepared from analytical-grade HCl (37%, Merck KGaA, Darmstadt, Germany). The solution pH was measured using a calibrated HANNA-211 pH meter. Dye concentrations were determined using a Shimadzu UV-1280 UV-vis spectrophotometer. The maximum absorbance wavelengths were determined separately for Trypan Blue and Ponceau S as *λ*_max_ = [593 nm] and *λ*_max_ = [525 nm], respectively. Calibration curves were prepared for each dye within the concentration range used in the experiments, and residual dye concentrations were calculated from the corresponding calibration equations. Blank and control measurements were included to minimize analytical uncertainty. Dye solutions without adsorbent were used to assess potential photolytic or time-dependent changes in concentration, while adsorbent suspensions without dye were used to evaluate any background absorbance or turbidity contributions after solid–liquid separation. When dilution was required before UV-vis analysis, the measured concentrations were corrected using the corresponding dilution factor.

### 2.2. Synthesis of Sr_2_Ni(BO_3_)_2_ adsorbent

The NiB adsorbent was synthesized by a combustion-assisted solid-state route using strontium nitrate, nickel nitrate hexahydrate, boric acid, and urea as precursors. The synthesized material was abbreviated as NiB throughout the manuscript. The precursor chemicals were Sr(NO_3_)_2_ (Sigma-Aldrich, ≥99.0%), Ni(NO_3_)_2_·6H_2_O (Sigma-Aldrich, ≥97.0%), H_3_BO_3_ (Sigma-Aldrich, ≥99.5%), and CO(NH_2_)_2_ (Sigma-Aldrich, ≥99.0%). All reagents were used as received without further purification.

The precursor molar ratio was adjusted according to the target Sr_2_Ni(BO_3_)_2_ composition. Briefly, 1.2696 g of Sr(NO_3_)_2_, 0.8724 g of Ni(NO_3_)_2_·6H_2_O, 0.3894 g of H_3_BO_3_, and 0.7206 g of CO(NH_2_)_2_ were accurately weighed using an analytical balance. These quantities corresponded approximately to the stoichiometric precursor ratio required to obtain about 1.05 g of NiB. The overall precursor reaction can be represented schematically as follows:2Sr(NO_3_)_2_ + Ni(NO_3_)_2_·6H_2_O + 2H_3_BO_3_ + 4CO(NH_2_)_2_ → Sr_2_Ni(BO_3_)_2_ + gaseous products

The weighed precursors were transferred into a porcelain capsule and dissolved in 50 mL of deionized water. The solution was heated on a hot plate at 100 °C until the water evaporated. The resulting dry precursor mixture was ground and homogenized. The powder was then pre-calcined at 500 °C for 15 min. After cooling, the material was crushed again in a porcelain mortar to improve homogeneity. The final powder was placed in an alumina boat and calcined at 850 °C for 6 h in a furnace. After calcination, the product was cooled to room temperature, ground, and stored in a clean, dry container until characterization and adsorption experiments.

### 2.3. Characterization of the adsorbent

#### 2.3.1. X-ray diffraction analysis

X-ray diffraction analysis was performed to determine the crystalline phase of the synthesized NiB adsorbent. XRD patterns were recorded using Cu Kα radiation on an Bruker AXS D8 Advance X-ray diffractometer. The obtained diffraction peaks were compared with the crystallographic reference card no. 00-197-1617 to verify the formation of the NiB phase and to identify possible secondary or impurity phases. XRD analysis was used primarily for phase confirmation and crystallinity assessment.

#### 2.3.2. Fourier-transform infrared spectroscopy

FTIR analysis was performed to identify the main surface functional groups of NiB and to evaluate potential spectral changes upon dye adsorption. FTIR spectra were recorded using an Thermo Fisher Scientific Nicolet iS5 FTIR spectrometer in the range of 4000–650 cm^−1^. Spectra were collected for the pristine NiB adsorbent and for NiB samples after adsorption with Trypan Blue and Ponceau S. The main vibrational regions associated with B–O, Ni–O, hydroxyl-related groups, and dye-derived sulfonate/aromatic structures were examined. Shifts or changes in band intensity after adsorption were used as evidence of possible interactions between the dye molecules and the NiB surface.

#### 2.3.3. SEM-EDX analysis

The surface morphology and elemental composition of NiB was examined using scanning electron microscopy (SEM; FEI Quanta FEG 250, operated at 10 kV) coupled with energy-dispersive X-ray spectroscopy (EDX; Octane Pro, EDAX, AMETEK, Inc.). Prior to SEM imaging, all samples were coated with a thin 10 nm Au/Pd layer using a Cressington sputter coater (108 Auto, Ted Pella, Inc.).

### 2.4. General batch adsorption procedure

Batch adsorption experiments were carried out separately for Trypan Blue and Ponceau S. Unless otherwise stated, 500 mL (0.5 L) of dye solution was transferred into appropriately sized Erlenmeyer flasks, and 0.1 g of NiB was added. Under these conditions, the adsorbent dose was 0.2 g L^−1^. The main experimental conditions were an initial dye concentration of 10 mg L^−1^, an agitation speed of 250 rpm, and a temperature of 25 °C. The solution pH, contact time, adsorbent dose, agitation speed, temperature, and initial dye concentration were varied depending on the experimental design described below.

The suspensions were agitated in a ZHICHENG ZHWY-200B orbital shaker. At the end of each adsorption experiment, the solid phase was separated from the liquid phase using a Sigma D-37520 centrifuge at 4000 rpm for 10 min. The residual dye concentration in the supernatant or filtrate was determined by UV-vis spectrophotometry. Adsorption experiments were performed in triplicate. The removal efficiency was calculated using eqn (1):1
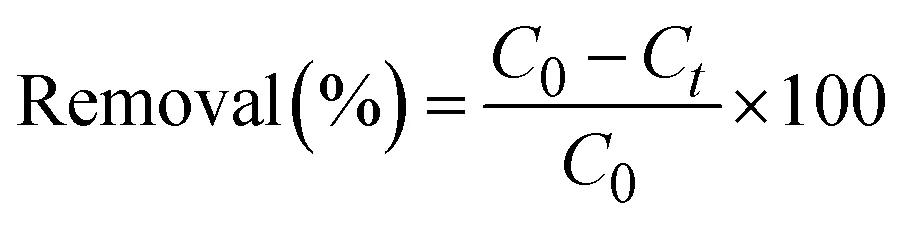


The adsorption capacity at time *t* and at equilibrium was calculated using eqn (2) and (3):2
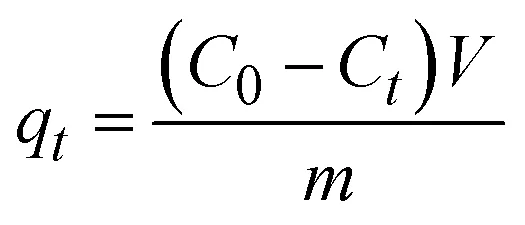
3
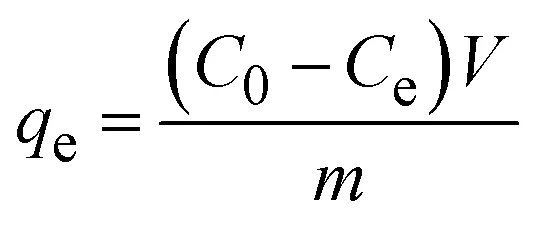
where *C*_0_ is the initial dye concentration (mg L^−1^), *C*_*t*_ is the dye concentration at time *t* (mg L^−1^), *C*_e_ is the equilibrium dye concentration (mg L^−1^), *V* is the solution volume (L), *m* is the adsorbent mass (g), *q*_*t*_ is the adsorption capacity at time *t* (mg g^−1^), and *q*_e_ is the equilibrium adsorption capacity (mg g^−1^). All adsorption capacity calculations were performed using the actual solution volume and adsorbent mass applied in each experiment.

### 2.5. Effect of solution pH

The effect of solution pH on the adsorption of Trypan Blue and Ponceau S was evaluated at pH 3.01, 7.50, 8.20, 10.20, and 11.60. The initial dye concentration, adsorbent mass, solution volume, agitation speed, and temperature were kept constant at 10 mg L^−1^, 0.1 g, 500 mL, 250 rpm, and 25 °C, respectively. The contact time was set to 30 minute. After adsorption, the residual dye concentration was measured, and the pH-dependent removal efficiency and adsorption capacity were calculated.

### 2.6. Effect of adsorbent dose

The effect of adsorbent dose was investigated using NiB masses of 0.05, 0.10, 0.20, 0.40, and 0.50 g in a 500 ml dye solution. These masses corresponded to adsorbent doses of 0.10, 0.20, 0.40, 0.80, and 1.00 g L^−1^, respectively. The initial dye concentration was maintained at 10 mg L^−1^. The pH, contact time, temperature, and agitation speed were set to 7.37 (Trypan Blue) and 7.52 (Ponceau S), 30 minutes, 25 °C, and 250 rpm, respectively. Because the adsorbent mass varied in this experiment, *q*_e_ values were calculated using the actual adsorbent mass applied at each experimental point.

### 2.7. Effect of agitation speed

The effect of agitation speed on dye adsorption was examined at 100, 150, 200, 250, and 300 rpm. These experiments were conducted using a 10 mg per L dye solution, 0.1 g NiB, and a 500 mL solution volume. pH, contact time, dose, and temperature were kept constant at 7.37 pH, 60 min, 0.1 g, and 25 °C for Trypan Blue, while for Ponceau S, they were kept constant at 7.52 pH, 60 min, 0.1 g, and 25 °C. The term agitation speed was used because the experiments were performed in an orbital shaker.

### 2.8. Contact time and kinetic experiments

The adsorption kinetics of Trypan Blue and Ponceau S onto NiB were investigated by sampling at different contact times. The selected contact times were 0, 15, 30, 60, 90, and 120 min. The initial dye concentration, adsorbent mass, and solution volume were 10 mg L^−1^, 0.1 g, and 500 mL, respectively. Under these conditions, the adsorbent dose corresponded to 0.2 g L^−1^. The pH, temperature, and agitation speed were kept constant at pH 7.37 for Trypan Blue and 7.52 for Ponceau S, 25 °C, and 250 rpm, respectively. The *q*_*t*_ values obtained at different contact times were used for kinetic modeling.

The kinetic data were evaluated using pseudo-first-order, pseudo-second-order, Elovich, intraparticle diffusion, and Boyd models. The non-linear forms of the pseudo-first-order, pseudo-second-order, Elovich, and intraparticle diffusion models were fitted directly to the original *q*_*t*_–*t* data by minimizing the residuals between the experimental and calculated *q*_*t*_ values. Boyd analysis was performed separately to evaluate the possible contribution of film diffusion and particle diffusion to the overall adsorption rate. This approach was adopted to reduce the potential bias associated with linear transformation of kinetic equations and to provide a more reliable comparison of kinetic model performance. The kinetic equations used in this study are summarized in Table 1.

**Table 1:** Kinetic and isotherm model equations used for the adsorption of Trypan Blue and Ponceau S onto NiB^a^

Model	Linear form	Non-linear form
Pseudo-first-order (PFO)	ln(*q*_e_ − *q*_*t*_) = ln *q*_e_ − *k*_1_*t*	*q*_*t*_ = *q*_e_(1 − e^−*k*_1_*t*^)
Pseudo-second-order (PSO)	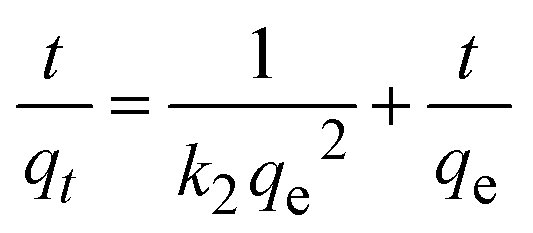	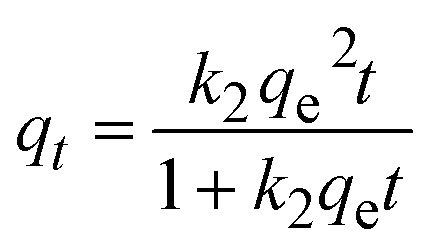
Elovich	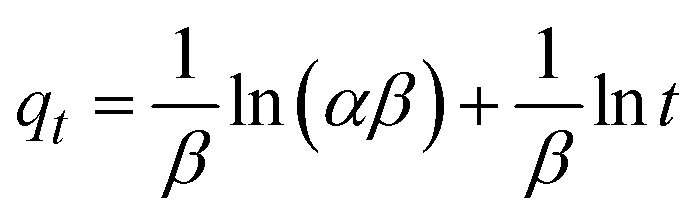	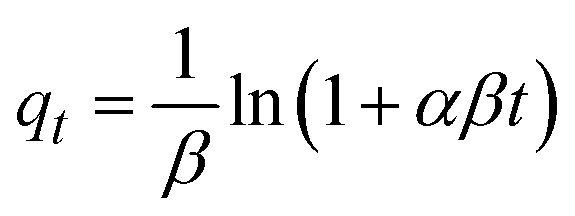
Intraparticle diffusion (ID)	*q*_*t*_ = *k*_id_*t*^1/2^ + *C*	—
Boyd	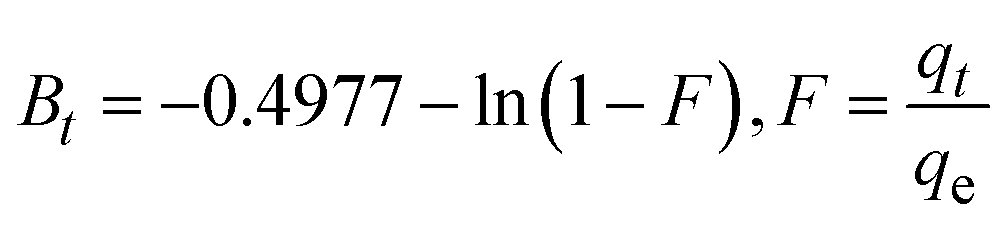	—
Langmuir	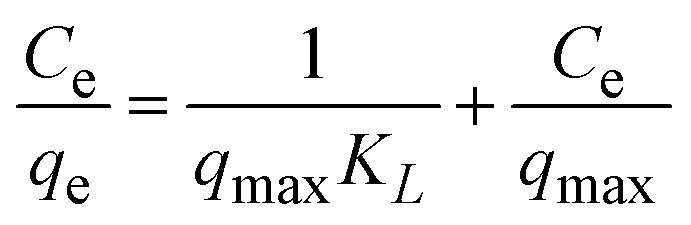	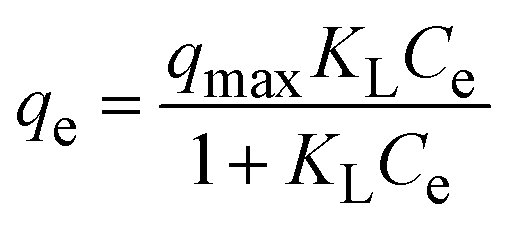
Freundlich	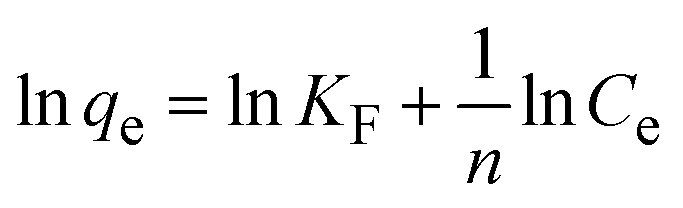	*q*_e_ = *K*_F_*C*_e_^1/*n*^
Temkin	*q*_e_ =*B*_*T*_* *ln *A*_*T*_ + *B*_*T*_ ln *C*_e_	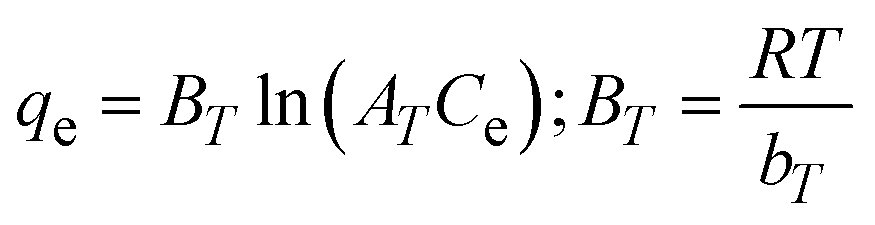
Sips	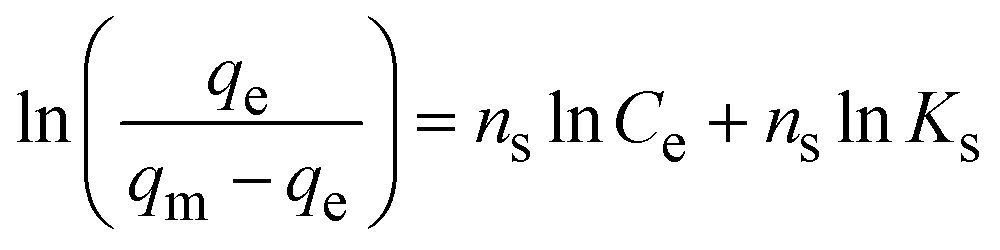	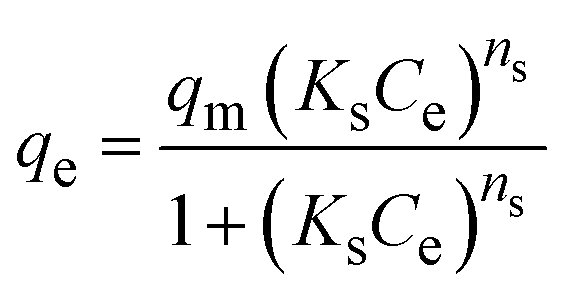
Redlich–Peterson	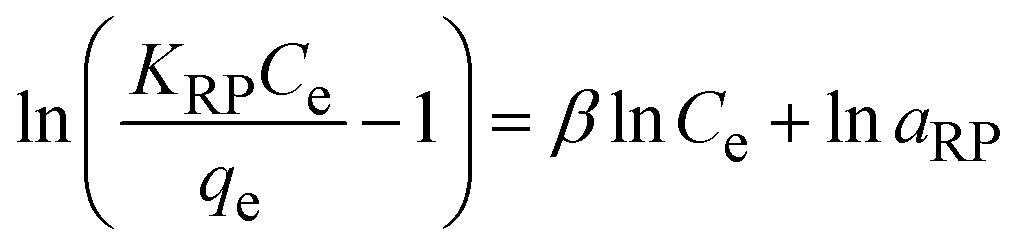	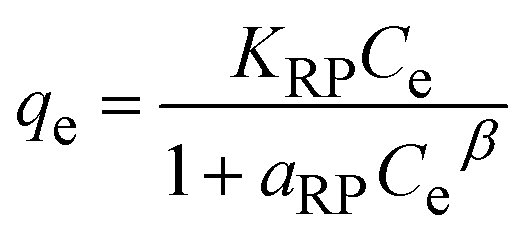
Dubinin–Radushkevich	ln *q*_e_ = ln *q*_m_ − *K*_DR_*ε*^2^	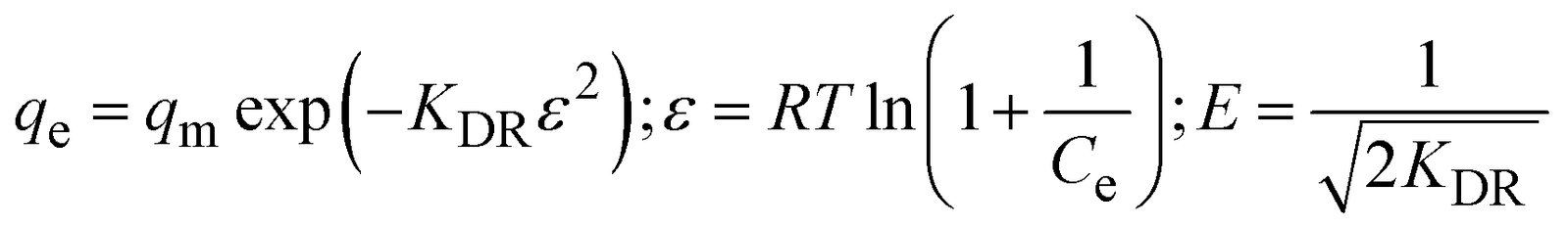

a The non-linear forms of the kinetic and isotherm models were used for final parameter estimation and model comparison. Linearized equations, where applicable, were considered only for preliminary visualization or comparison and were not used as the primary basis for final model selection.

In these equations, *q*_*t*_ and *q*_e_ are the adsorption capacities at time *t* and equilibrium, respectively, expressed in mg g^−1^; *k*_1_ is the pseudo-first-order rate constant (min^−1^); *k*_2_ is the pseudo-second-order rate constant (g mg^−1^ min^−1^); *α* is the initial adsorption rate in the Elovich model (mg g^−1^ min^−1^); *β* is the Elovich constant related to desorption energy (g mg^−1^); *k*_id_ is the intraparticle diffusion rate constant (mg g^−1^ min^−1/2^); *C* is the intercept associated with boundary layer effects; *F* is the fractional adsorption; and *B*_*t*_ is the Boyd parameter. *C*_e_ is the equilibrium dye concentration (mg L^−1^), and *q*_e_ is the equilibrium adsorption capacity (mg g^−1^). *q*_max_ is the Langmuir maximum monolayer adsorption capacity (mg g^−1^), and *K*_L_ is the Langmuir constant (L mg^−1^). *K*_f_ is the Freundlich adsorption capacity constant, and 1/*n* is the Freundlich adsorption intensity or heterogeneity parameter. *A*_T_ is the Temkin binding constant (L g^−1^), *B*_T_ is the Temkin constant, and *b*_T_ is the Temkin constant related to adsorption heat. *q*_m_ represents the theoretical adsorption capacity in the Dubinin–Radushkevich and Sips models (mg g^−1^). *K*_DR_ is the Dubinin–Radushkevich constant, *ε* is the Polanyi potential, *E* is the mean adsorption energy, *R* is the universal gas constant, and *T* is the absolute temperature (*K*). In the Dubinin–Radushkevich calculations, concentration units were kept consistent throughout. *K*_s_ is the Sips affinity constant (L mg^−1^), and *n*_s_ is the Sips heterogeneity factor. *K*_RP_ is the Redlich–Peterson constant related to adsorption capacity (L g^−1^), *a*_RP_ is the Redlich–Peterson constant related to the affinity of binding sites (*L*_β_ mg^−1^), and *β* is the Redlich–Peterson exponent reflecting adsorption intensity. Because Sips and Redlich–Peterson are three-parameter isotherm models, their fitting results were interpreted with caution, particularly given the limited number of equilibrium data points. Linearized equations, where applicable, were used only for preliminary visualization or comparison and were not used as the primary basis for final model selection.

### 2.9. Equilibrium adsorption and isotherm modeling

Equilibrium adsorption experiments were conducted to evaluate the dye-loading behavior of NiB and the relationship between equilibrium dye concentration and adsorption capacity. Initial dye concentrations of 10, 25, 30, 50, and 100 mg L^−1^ were used for both Trypan Blue and Ponceau S. In all isotherm experiments, the adsorbent mass and solution volume were fixed at 0.1 g and 500 mL, respectively. The pH, agitation speed, temperature, and contact time were maintained at pH 7.37 for Trypan Blue and pH 7.52 for Ponceau S, 250 rpm, 25 °C, and 60 min, respectively.

The experimental equilibrium data were fitted using the non-linear forms of the Langmuir, Freundlich, Temkin, Dubinin–Radushkevich (D–R), Sips, and Redlich–Peterson (R–P) isotherm models (Table 1). The non-linear fitting approach was selected as the primary method for parameter estimation because it allows direct fitting of the original *C*_e_–*q*_e_ data without transformation of the experimental variables. This approach minimizes the potential distortion of the error structure that may occur when isotherm equations are linearized.

The non-linear model parameters were obtained by least-squares minimization of the residuals between the experimental and calculated *q*_e_ values. Model suitability was evaluated using the coefficient of determination (*R*^2^) together with multiple error functions, including the sum of squared errors (SSE), root mean square error (RMSE), chi-square error (*χ*^2^), average relative error (ARE), and the HYBRID error function, as described in Section 2.12. Linearized equations, where applicable, were used only for preliminary comparison and were not considered as the primary basis for final model selection. Because the equilibrium data did not show a clear saturation plateau within the tested concentration range, model-derived *q*_max_ values, particularly those obtained from Langmuir-type models, were interpreted cautiously as apparent estimates rather than definitive maximum adsorption capacities.

### 2.10. Effect of temperature and thermodynamic evaluation

The effect of temperature on the adsorption of Trypan Blue and Ponceau S was examined at 25, 30, 35, 40, and 45 °C, corresponding to 298.15, 303.15, 308.15, 313.15, and 318.15 K, respectively. The initial dye concentration, adsorbent mass, and solution volume were fixed at 10 mg L^−1^, 0.1 g, and 500 mL, respectively. The pH, contact time, and agitation speed were maintained at 7.37 for Trypan Blue and 7.52 for Ponceau S, for 60 min at 250 rpm, respectively. For thermodynamic evaluation, an apparent concentration-ratio-based equilibrium constant (*K*_app_) was calculated from the initial and equilibrium dye concentrations, as shown in Table 2.

**Table 2:** Thermodynamic equations used for Trypan Blue and Ponceau S adsorption onto NiB^a^

Parameter	Equation
Dimensionless equilibrium constant	*K*_app_ = (*C*_0_ − *C*_e_)/*C*_e_
Standard Gibbs free energy change	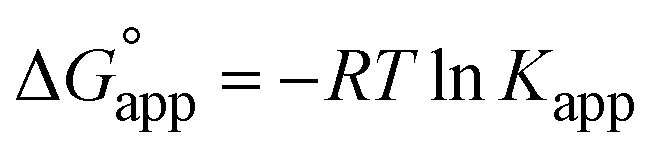
Van't Hoff equation	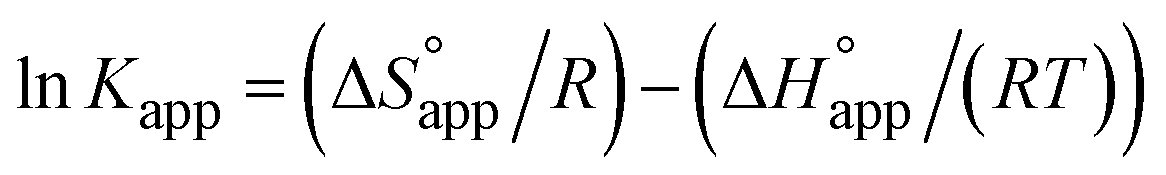

a where *K*_app_ is the apparent concentration-ratio-based equilibrium constant, *C*_0_ is the initial dye concentration (mg L^−1^), *C*_e_ is the equilibrium dye concentration (mg L^−1^), *R* is the universal gas constant (8.314 J mol^−1^ K^−1^), *T* is the absolute temperature (*K*), 
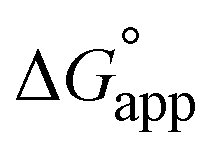
 is the apparent Gibbs free energy change, 
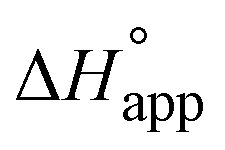
 is the apparent enthalpy change, and 
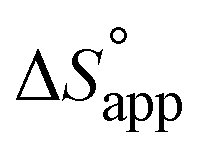
 is the apparent entropy change. Because *K*_app_ was calculated from a concentration ratio, the thermodynamic parameters were evaluated as comparative indicators of adsorption spontaneity and temperature dependence rather than as absolute standard-state thermodynamic constants.

### 2.11. Regeneration and reuse experiments

Regeneration and reuse experiments were conducted to evaluate the reusability of NiB after Trypan Blue and Ponceau S adsorption. Dye-loaded NiB was separated from the solution using a Sigma D-37520 centrifuge at 4000 rpm for 10 min., washed gently with deionized water to remove loosely attached dye, and subjected to chemical or thermal regeneration. For chemical regeneration, dye-loaded NiB was treated with 0.1 M HCl for 30 min at room temperature under agitation at 250 rpm. For chemical regeneration, the dye-loaded NiB was treated under the optimal adsorption conditions. After acid treatment, the adsorbent was separated, washed with deionized water until the pH approached neutrality, dried at 60 °C, and reused in the next adsorption cycle. For thermal regeneration, dye-loaded NiB was heated at 500 °C for 30 min. After cooling to room temperature, the regenerated adsorbent was reused in a new adsorption cycle under the same adsorption conditions. Regeneration performance was monitored over nine consecutive adsorption–regeneration cycles. At the end of each cycle, the residual dye concentration was measured, and removal efficiency was calculated. Reuse performance was expressed as the remaining removal efficiency and, where applicable, as the percentage of the initial adsorption performance retained after each cycle.

### 2.12. Error analysis and model validation

The suitability of the kinetic and isotherm models was evaluated using *R*^2^ and multiple error metrics. This approach was adopted because model selection based only on *R*^2^ may lead to misleading conclusions, particularly when linearized model forms distort the original error structure. For kinetic modeling, the non-linear forms of the pseudo-first-order, pseudo-second-order, Elovich, and intraparticle diffusion models were fitted directly to the experimental *q*_*t*_–*t* data. The fitting procedure was performed by least-squares minimization of the residuals between the experimental and model-calculated *q*_*t*_ values. Boyd analysis was evaluated separately as a diffusion diagnostic tool to assess the possible contribution of film diffusion and particle diffusion to the overall adsorption rate. Therefore, *q*_*t*_-based error functions were not applied to the Boyd model.

For isotherm modeling, the non-linear forms of the Langmuir, Freundlich, Temkin, Dubinin–Radushkevich, Sips, and Redlich–Peterson models summarized in Table 1 were fitted directly to the experimental *C*_e_–*q*_e_ data. The fitting procedure was performed by least-squares minimization of the residuals between the experimental and model-calculated *q*_e_ values. All non-linear least-squares fitting procedures were performed using a Python-based computational workflow. Calculations were carried out using Python 3.11 with the NumPy and SciPy libraries, and the model parameters were obtained by minimizing the residuals between the experimental adsorption capacities and the values calculated from the corresponding non-linear model equations.

Model suitability was assessed using the coefficient of determination (*R*^2^), sum of squared errors (SSE), root mean square error (RMSE), chi-square error (*χ*^2^), average relative error (ARE), and HYBRID error function. Lower SSE, RMSE, *χ*^2^, ARE, and HYBRID values, together with higher *R*^2^ values, were considered to indicate better model performance. The calculated goodness-of-fit and error-function values were reported together with the corresponding non-linear isotherm and kinetic model parameters.

### 2.13. Statistical treatment and data presentation

All experimental data were processed using a Python-based computational workflow in Python 3.11 with the NumPy and SciPy libraries. The original experimental *C*_e_–*q*_e_ and *q*_*t*_–*t* data were fitted directly to the corresponding non-linear isotherm and kinetic equations summarized in Table 1. Non-linear model parameters were estimated by least-squares minimization of the residuals between the experimental and model-calculated adsorption capacities. Linearized plots, where applicable, were used only for supplementary or preliminary visual comparison and were not used as the primary basis for model selection. Results were presented as mean values, and model adequacy was evaluated using *R*^2^ together with SSE, RMSE, *χ*^2^, ARE, and HYBRID error functions. Figures were prepared using Matplotlib in Python.

## 3 Results and discussion

### 3.1. Characterization of the Sr_2_Ni(BO_3_)_2_ adsorbent

The structural and surface properties of the synthesized NiB adsorbent, hereafter abbreviated as NiB, were evaluated by X-ray diffraction (XRD) and Fourier-transform infrared spectroscopy (FTIR). The XRD pattern of NiB exhibited several sharp and well-resolved diffraction peaks within the 2*θ* range of approximately 10–50° (Fig. 1a), indicating that the synthesized material had a crystalline rather than amorphous structure. The diffraction peaks were indexed by comparison with the crystallographic reference card no. 00-197-1617, confirming the formation of the NiB phase. The indexed pattern was consistent with a monoclinic crystal system with the *C*2/*m* space group. The intense reflections observed at approximately 28–31° further supported the formation of an ordered Sr–Ni–borate crystalline framework.

**Fig. 1:**
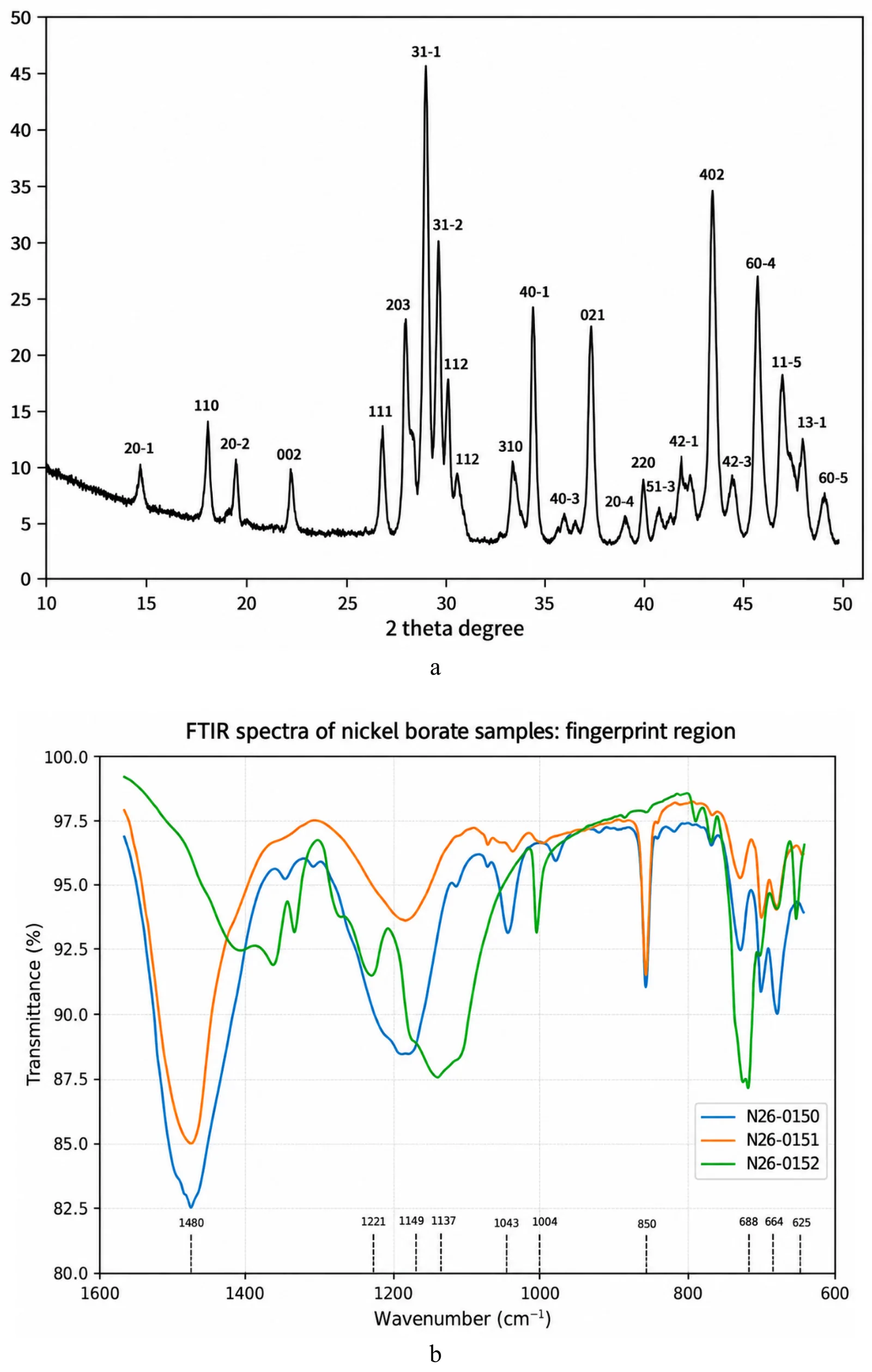
Characterization of the Sr_2_Ni(BO_3_)_2_ adsorbent: (a) XRD pattern indexed according to crystallographic reference card no. 00-197-1617, confirming the monoclinic Sr_2_Ni(BO_3_)_2_ phase; and (b) comparative FTIR spectra of NiB before adsorption and after Trypan Blue and Ponceau S adsorption.

FTIR analysis was performed to identify the main vibrational features of NiB and to examine possible spectral changes after Trypan Blue and Ponceau S adsorption (Fig. 1b). In the spectrum of the pristine adsorbent, the bands observed at approximately 1455, 1175, 1042, 857, 732, 705, and 684 cm^−1^ were mainly associated with B–O vibrations, Ni–O-related vibrations, and the borate skeletal structure. After adsorption, changes in band intensity and/or position were observed, particularly in the 1200–1000 cm^−1^ and 700–900 cm^−1^ regions. These changes may be related to interactions between dye-derived functional groups and the NiB surface. The 1200–1000 cm^−1^ region is relevant to B–O vibrations and may overlap with sulfonate-related vibrations of the dye molecules, whereas the 700–900 cm^−1^ region is associated with Ni–O and borate-framework vibrations. Therefore, the FTIR results suggest that dye retention on the NiB surface was not solely due to weak physical attachment but may also involve specific interactions between the dye's functional groups and surface sites. However, because FTIR provides indirect evidence of surface interactions, the proposed mechanism was interpreted alongside pH behavior, kinetic modeling, thermodynamic trends, and regeneration results.

SEM-EDX analysis was further used to evaluate the morphological characteristics and elemental composition of pristine NiB and dye-loaded NiB samples. SEM images of the pristine NiB adsorbent showed irregularly shaped, agglomerated particles with a rough, heterogeneous surface morphology (Fig. 2a–d). This type of surface structure may provide multiple contact points and accessible surface domains for dye retention. After Trypan Blue adsorption, the NiB surface appeared more covered and compact, suggesting that dye molecules were deposited on the external surface and within surface irregularities (Fig. 2e–h). A similar surface modification was observed after Ponceau S adsorption, where the particles exhibited increased surface coverage and a more aggregated appearance compared with the pristine adsorbent (Fig. 2i–l). These morphological changes support the occurrence of dye accumulation on the NiB surface after adsorption.

**Fig. 2:**
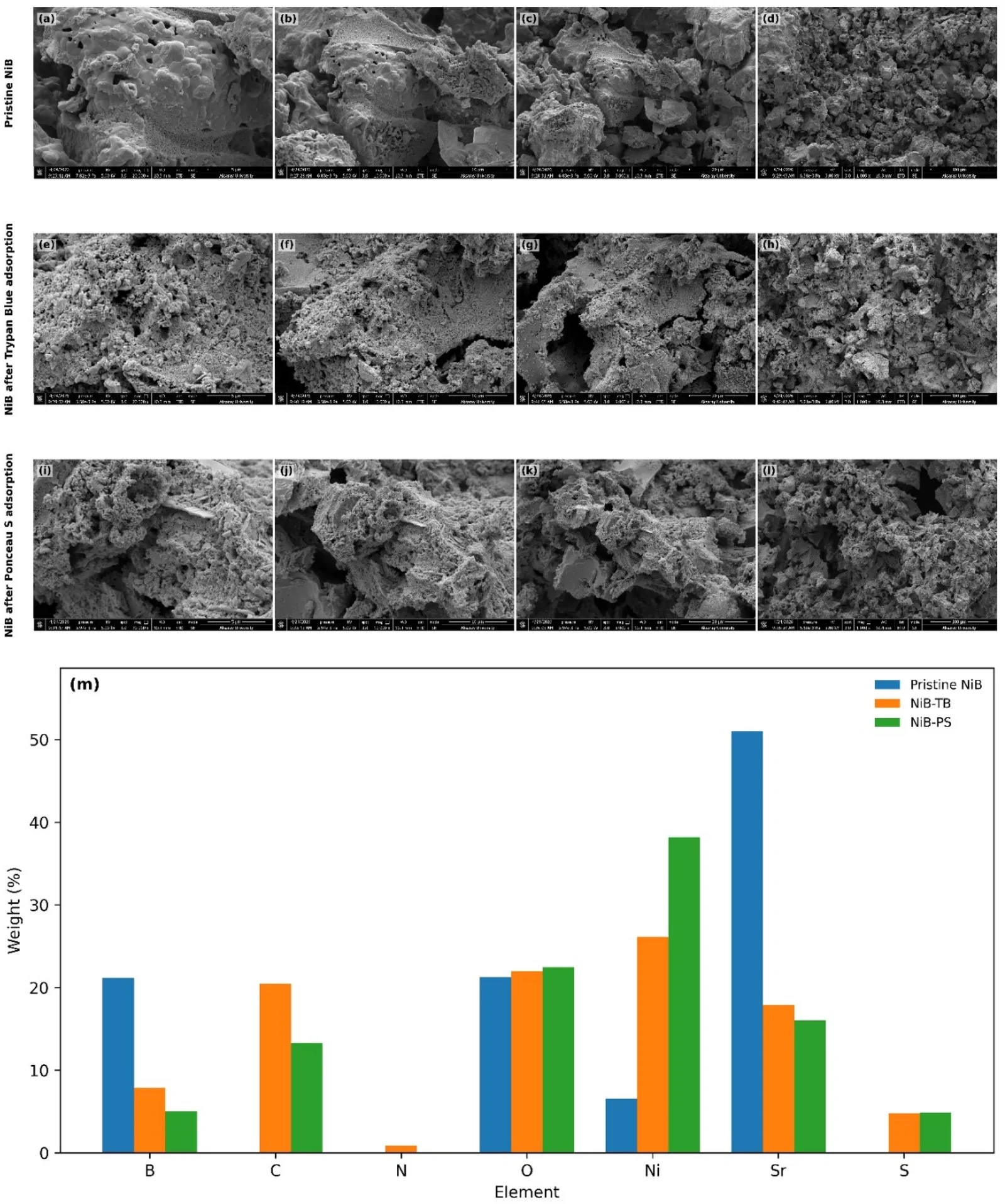
SEM-EDX characterization of Sr_2_Ni(BO_3_)_2_/NiB before and after dye adsorption: (a–d) SEM images of pristine NiB before adsorption, (e–h) SEM images of NiB after Trypan Blue adsorption, (i–l) SEM images of NiB after Ponceau S adsorption, and (m) comparative EDX-based elemental composition of pristine NiB, NiB-TB, and NiB-PS samples.

The EDX results provided additional evidence for dye retention on the NiB surface. The pristine NiB sample mainly contained B, O, Ni, and Sr, confirming the inorganic composition of the Sr–Ni–borate framework (Fig. 2m). In contrast, after Trypan Blue adsorption, C, N, and S signals were detected in addition to the structural elements of NiB (Fig. 2m). The presence of C and S is particularly important because Trypan Blue contains an aromatic carbon skeleton and sulfonate groups. Similarly, after Ponceau S adsorption, C and S signals were observed on the adsorbent surface, indicating the retention of the sulfonated dye molecules (Fig. 2m). The EDX spectra showed that S was detected after both dye adsorption processes, supporting the involvement of sulfonate-containing dye structures in surface attachment. The appearance of dye-derived elements after adsorption, together with the partial change in the relative proportions of B, Ni, and Sr, can be attributed to the formation of an adsorbed organic dye layer that partially masked the inorganic NiB surface.

These SEM-EDX observations are consistent with the FTIR results and adsorption performance. The surface coverage observed in SEM images and the appearance of C, N, and/or S signals in the EDX spectra indicate that Trypan Blue and Ponceau S were retained on the NiB surface. Similar interpretations have been reported for borate-based and metal oxide/inorganic adsorbents, where dye-derived elemental signals detected after adsorption have been used as supporting evidence for the surface accumulation of anionic dye molecules.^8,18,23^ However, EDX analysis provides semi-quantitative and surface-localized information, and light elements such as B may be detected with limited accuracy. Therefore, SEM-EDX findings were evaluated as complementary evidence together with XRD, FTIR, pH-dependent adsorption behavior, kinetic modeling, and thermodynamic evaluation, rather than as independent proof of the adsorption mechanism.

### 3.2. Adsorption performance of NiB toward Trypan Blue and Ponceau S

The adsorption performance of the synthesized NiB adsorbent was evaluated for the removal of two sulfonated anionic dyes, Trypan Blue and Ponceau S, from aqueous solutions. Under the main experimental conditions, the initial dye concentration was 10 mg L^−1^, the solution volume was 500 mL, and the adsorbent mass was 0.1 g, corresponding to an adsorbent dose of 0.2 g L^−1^. High removal efficiencies were obtained for both dyes, particularly under near-neutral conditions, at 250 rpm agitation and 25 °C. The differences observed between Trypan Blue and Ponceau S cannot be attributed only to their anionic character. Molecular size, sulfonate group density and distribution, aromatic structure, hydration shell, and molecular orientation relative to the adsorbent surface may also affect their adsorption behavior. The NiB surface contains Ni–O, B–O, and hydroxyl-related sites that may contribute to dye retention *via* electrostatic attraction, hydrogen bonding, Lewis acid–base interactions, and surface complexation. Similar combined interaction pathways have been reported for aromatic pollutants and dyes adsorbed onto GO/SiO_2_ hybrids, graphene-based sorbents, porous organic frameworks, and inorganic or hydroxylated adsorbent surfaces.^24–28^ In addition, previous studies on Trypan Blue adsorption have shown that physical adsorption, hydrogen bonding, and surface-orientation effects can contribute simultaneously to dye retention, supporting the interpretation that the different removal behaviors of Trypan Blue and Ponceau S are related to both dye structure and NiB surface chemistry.^16,29^

### 3.3. Effect of operating parameters on dye adsorption

#### 3.3.1. Effect of contact time

Contact time influences dye transport from the bulk solution to the adsorbent surface, the occupation of available surface sites, and possible diffusion into internal or interparticle domains. In this study, the adsorption of Trypan Blue and Ponceau S was evaluated over 15–120 min (Fig. 3a). For both dyes, removal efficiency increased rapidly during the first 60 min. Trypan Blue removal increased from 78.95% at 15 min to 93.72% at 60 min, while the adsorption capacity increased from 39.48 to 46.86 mg g^−1^. A similar trend was observed for Ponceau S, with removal increasing from 70.57% at 15 min to 90.54% at 60 min, and an adsorption capacity of 45.27 mg g^−1^ at 60 min. The rapid initial uptake can be attributed to the high concentration gradient between the solution and the NiB surface and to the availability of initially accessible Ni–O, B–O, and hydroxyl-related sites. At this early stage, external film diffusion and surface interactions, such as electrostatic attraction, hydrogen bonding, and Lewis acid–base interactions, may jointly contribute to dye retention.^19,25,26,30^ After 60 min, the limited decrease or fluctuation in removal efficiency observed at 90 and 120 min may reflect the progressive occupation of high-affinity sites, the establishment of adsorption–desorption equilibrium, and limited intraparticle or interparticle diffusion. Minor variations at longer contact times may also be due to experimental variability during solid–liquid separation and UV-vis measurements. Similar multi-step kinetic behavior has been reported for dye adsorption on heterogeneous adsorbents and borate-based materials, where rapid initial adsorption is followed by a slower approach to equilibrium.^19–21,30,31^

**Fig. 3:**
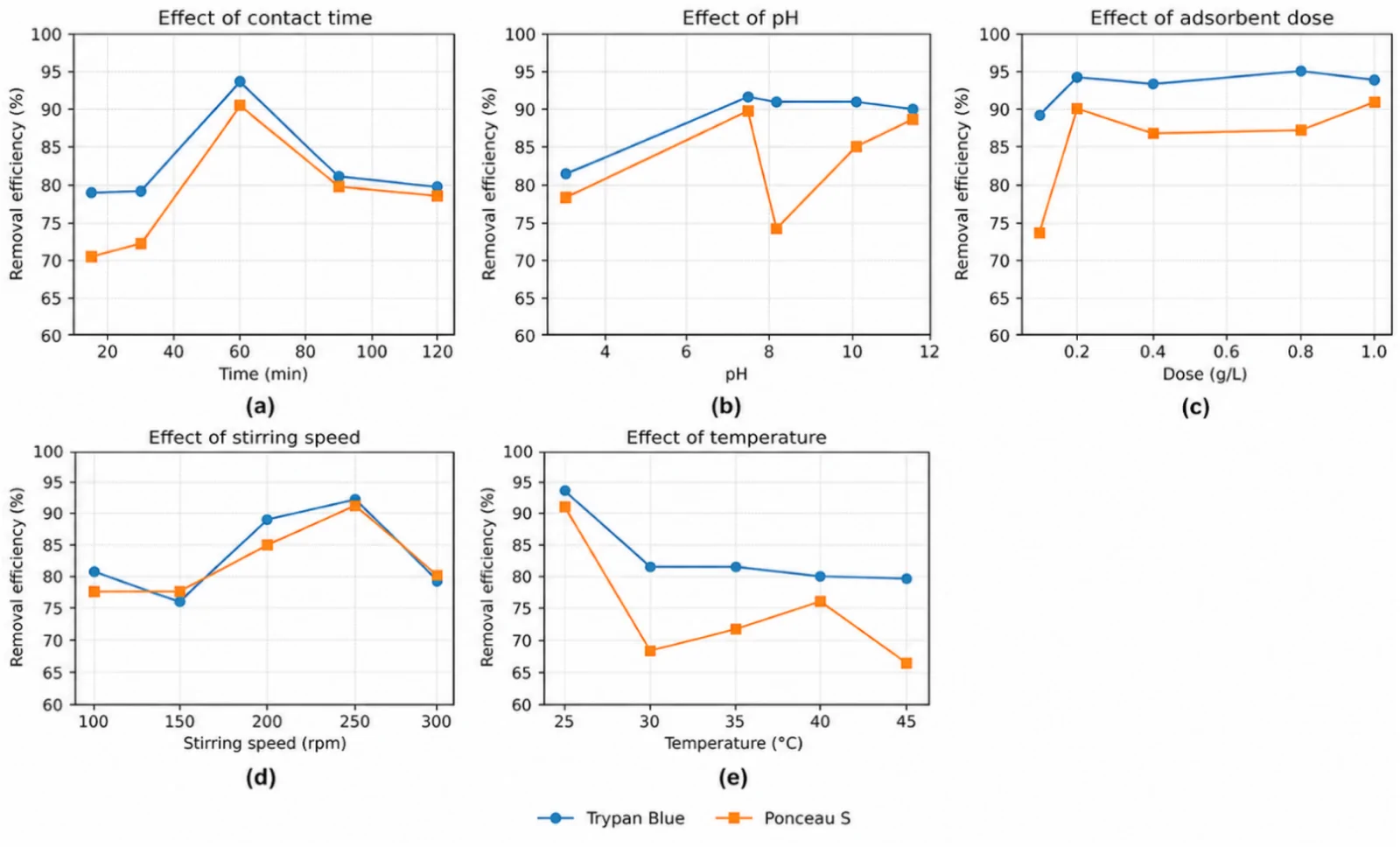
Effects of operating parameters on Trypan Blue and Ponceau S adsorption by NiB: (a) contact time, (b) solution pH, (c) adsorbent dose, (d) agitation speed, and (e) temperature. Experimental results are presented in comparative terms as removal efficiency (%).

#### 3.3.2. Effect of solution pH

Solution pH affects the protonation/deprotonation state of the adsorbent surface, the ionization behavior of dye molecules, and the strength of surface–adsorbate interactions. For anionic dyes such as Trypan Blue and Ponceau S, pH-dependent changes can influence electrostatic attraction, hydrogen bonding, and possible surface complexation. In the present study, Trypan Blue removal increased from 81.52% at pH 3.01 to 91.55% at pH 7.50 and remained high, between 89.99% and 90.99%, over the pH range of 8.20–11.60 (Fig. 3b). This behavior indicates that Trypan Blue adsorption was not restricted to acidic conditions and that NiB retained adsorption-active surface sites over a relatively broad pH range. For Ponceau S, removal efficiency increased from 78.35% at pH 3.01 to 89.72% at pH 7.50, decreased to 74.21% at pH 8.20, and then increased again to 85.13–88.66% at pH 10.20–11.60 (Fig. 3b). This more variable, pH-dependent response indicates that Ponceau S adsorption was more sensitive to surface charge conditions, sulfonate group position, molecular orientation, and possible competition with hydroxide ions than Trypan Blue was. Because direct pH_pzc_ or zeta potential measurements were not performed, the contribution of surface charge was evaluated indirectly based on the pH-dependent adsorption behavior. The maintenance of high removal efficiencies under neutral and alkaline conditions indicates that electrostatic attraction alone cannot fully account for adsorption. Instead, hydrogen bonding, Lewis acid–base interactions, coordinative interactions, surface complexation, and weak physical interactions likely contributed simultaneously. Similar interpretations have been reported for boron-containing, metal-oxide, and hydroxylated adsorbents, in which pH effects are governed by surface charge and functional-group-specific interactions.^17,18^

#### 3.3.3. Effect of adsorbent dose

The adsorbent dose determines the total number of available surface sites and the probability of contact between dye molecules and the solid phase. In this study, the NiB mass was varied between 0.05 and 0.50 g. Given a solution volume of 500 mL, these masses correspond to adsorbent doses of 0.10–1.00 g L^−1^. Because the adsorbent mass changed during the dose experiments, *q*_e_ values were calculated using the actual mass applied at each experimental point. For Trypan Blue, removal remained high across the tested dose range, with the highest efficiency of 95.05% observed at 0.40 g NiB (Fig. 3c). The highest unit adsorption capacity, however, was obtained at the lowest dose, reaching 89.14 mg g^−1^ at 0.05 g. For Ponceau S, removal increased from 73.55% at 0.05 g to 89.92% at 0.10 g, then to 90.96% at 0.50 g (Fig. 3c). This inverse relationship between removal efficiency and adsorption capacity per unit mass is commonly observed in batch adsorption systems. At a fixed initial dye concentration, increasing the adsorbent mass increases the total number of available sites, thereby enhancing removal efficiency. In contrast, *q*_e_ generally decreases at higher adsorbent doses because the same dye mass is distributed over a larger amount of adsorbent and because many sites remain unsaturated.

Similar dose-capacity behavior has been reported in various dye adsorption systems and is commonly attributed to active-site availability, surface heterogeneity, mass transfer, and incomplete saturation of adsorption sites at high adsorbent doses.^19,30,31^ The high *q*_e_ values obtained at low NiB doses may also be attributed to the contributions of Ni–O, B–O, and hydroxyl-related sites to dye retention *via* combined surface interactions.^17,26^

#### 3.3.4. Effect of agitation speed

Agitation speed affects external mass transfer, the thickness of the liquid film surrounding adsorbent particles, and the frequency of adsorbent–adsorbate contact. Trypan Blue removal increased from 80.81% at 100 rpm to 92.14% at 250 rpm but decreased to 78.93% at 300 rpm (Fig. 3d). Ponceau S followed a similar trend: removal remained close to 77% at 100–150 rpm, reached 91.21% at 250 rpm, and decreased to 80.14% at 300 rpm (Fig. 3d). At lower agitation speeds, a thicker boundary layer around NiB particles may restrict dye transport to accessible surface sites, thereby increasing external film diffusion resistance. Increasing the agitation speed to 250 rpm improved mass transfer and enhanced adsorbent–adsorbate contact, resulting in the highest removal efficiencies for both dyes. The decline observed at 300 rpm may be associated with particle redistribution, reduced contact stability, or partial detachment of weakly retained dye molecules under stronger hydrodynamic conditions. Therefore, 250 rpm was selected as the optimal agitation speed, providing sufficient mass transfer without a significant decrease in adsorption performance. Similar agitation-dependent adsorption behavior has been reported in other dye adsorption systems.^31,32^

#### 3.3.5. Effect of temperature and thermodynamic interpretation

Temperature influences adsorbate diffusion, the stability of surface interactions, and the thermodynamic direction of adsorption. In this study, the highest removal efficiency was obtained at 25 °C for both dyes (Fig. 3e). Trypan Blue removal decreased from 93.74% at 25 °C to 81.45% at 30 °C, then remained in the range of approximately 79.66–81.49% between 35 and 45 °C. Ponceau S removal decreased more sharply, from 90.96% at 25 °C to 68.31% at 30 °C, followed by limited fluctuations at higher temperatures. The decrease in removal efficiency with increasing temperature was consistent with the calculated negative Δ*H*° values, indicating that the adsorption process is exothermic. The negative Δ*G*° values, ranging from approximately −27.82 to −25.20 kJ mol^−1^ for Trypan Blue and from −26.84 to −23.40 kJ mol^−1^ for Ponceau S, indicate that adsorption occurred spontaneously at the tested temperatures (Table 3). The negative Δ*S*° values indicate a decrease in randomness at the solid–liquid interface, which may be associated with the organization of dye molecules on or near the NiB surface during adsorption. Because the thermodynamic parameters were calculated using an apparent concentration-ratio-based equilibrium constant, they were evaluated as comparative thermodynamic indicators rather than absolute standard-state constants. Similar trends, including negative Δ*G*°, negative Δ*H*°, and higher adsorption efficiency at lower temperatures, have been associated with spontaneous and exothermic dye adsorption in other adsorbent systems.^33–38^ The stronger adsorption at lower temperatures may reflect more stable dye–surface interactions involving Ni–O, B–O, and hydroxyl-related sites.^17,18^

**Table 3:** Apparent thermodynamic parameters for Trypan Blue and Ponceau S adsorption onto NiB

Parameter	Trypan blue	Ponceau S
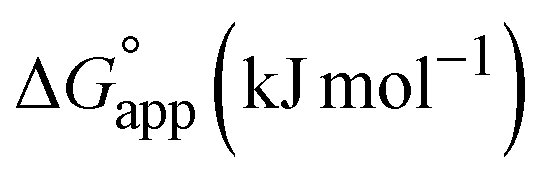	−27.82 to −25.20	−26.84 to −23.40
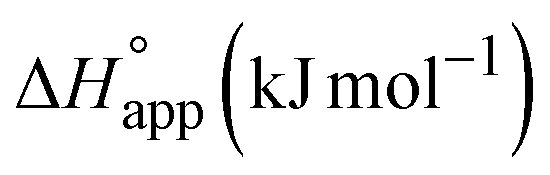	−44.32	−45.86
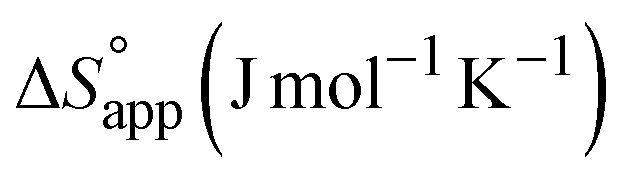	−59.07	−68.38

### 3.4. Equilibrium adsorption and isotherm evaluation

Equilibrium adsorption experiments were performed to evaluate the loading capacity and surface saturation tendency of NiB toward Trypan Blue and Ponceau S at different initial concentrations. The *q*_e_ values and removal efficiencies were calculated using the actual initial dye concentration, equilibrium dye concentration, solution volume, and adsorbent mass at each experimental point. The corrected isotherm data and corresponding removal efficiencies are presented in Fig. 4 and Table 4.

**Fig. 4:**
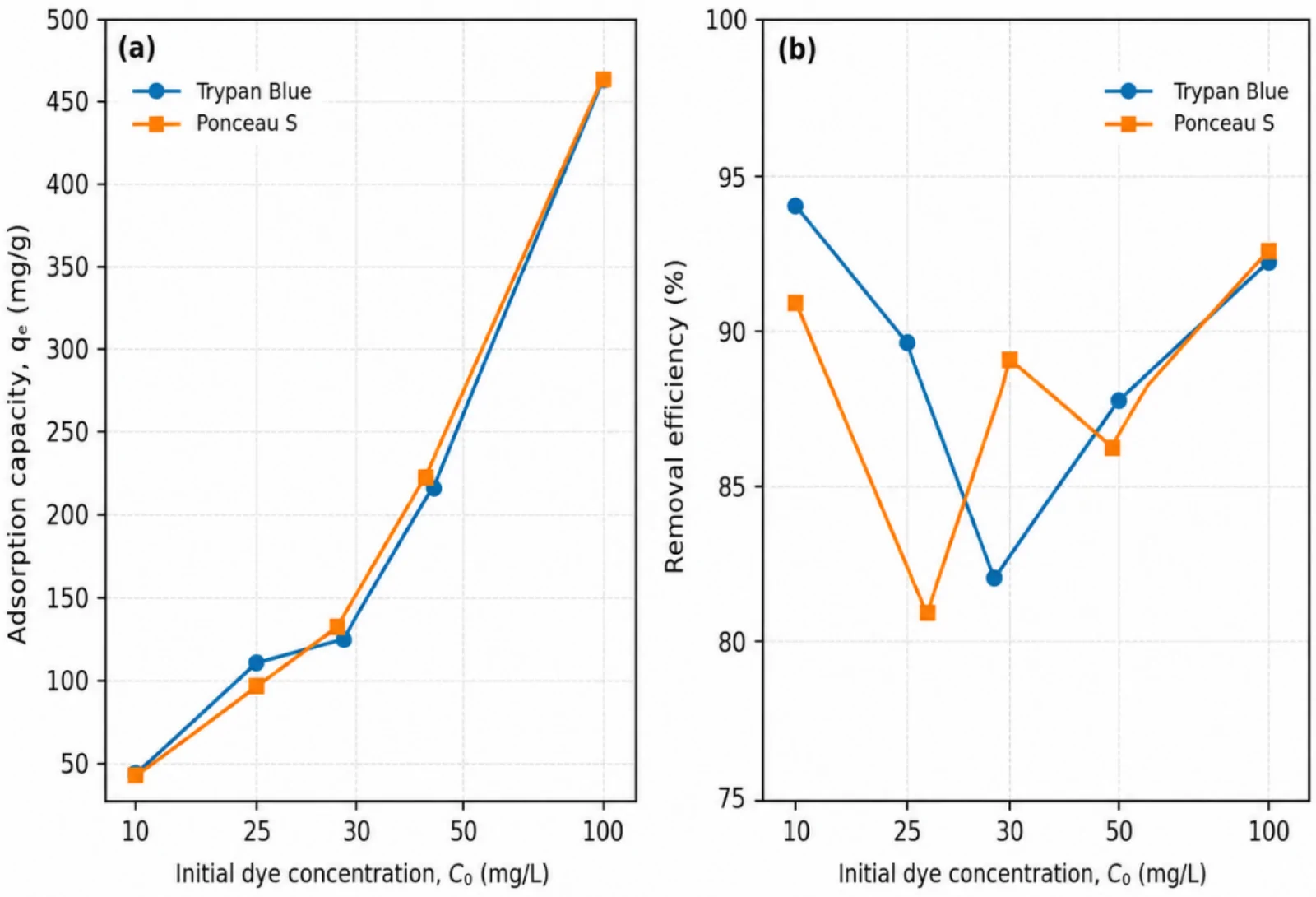
Corrected equilibrium adsorption data based on actual initial dye concentrations for Trypan Blue and Ponceau S adsorption onto NiB: (a) equilibrium adsorption capacity (*q*_e_) as a function of initial dye concentration (*C*_0_), and (b) removal efficiency as a function of initial dye concentration (*C*_0_).

**Table 4:** Non-linear isotherm parameters and error-function values for Trypan Blue and Ponceau S adsorption onto NiB

Dye	Model	Model parameters	*R* ^2^	SSE	RMSE	*χ* ^2^	ARE (%)	HYBRID
Trypan blue	Langmuir	*q*_max_ = 1.00 × 10^7^ mg g^−1^	0.674	34691.09	83.30	136.19	39.25	5695.84
		*K*_L_ = 4.48 × 10^−6^ L mg^−1^						
Ponceau S	Langmuir	*q*_max_ = 1.00 × 10^7^ mg g^−1^	0.643	38288.36	87.51	139.91	36.61	5723.94
		*K*_L_ = 4.39 × 10^−6^ L mg^−1^						
Trypan blue	Freundlich	*K*_f_ = 0.235	0.877	13116.51	51.22	65359.67	41.52	4777.22
		1/*n* = 3.787; *n* = 0.264						
Ponceau S	Freundlich	*K*_f_ = 6.50;	0.718	30232.88	77.76	427.52	48.90	5310.91
		1/*n* = 2.030; *n* = 0.493						
Trypan blue	Temkin	*A*_T_ = 1.74 L g^−1^	0.487	54599.30	104.50	565.03	59.66	8933.03
		*B*_T_= 110.743						
Ponceau S	Temkin	*A*_T_ = 1.10 L g^−1^	0.502	53413.99	103.36	2163.95	62.31	9302.70
		*B*_T_ = 137.718						
Trypan blue	Dubinin–Radushkevich	*q*_m_ = 2.58 × 10^3^ mg g^−1^	0.859	15051.67	54.87	2.21 × 10^15^	43.31	5361.61
		*K*_DR_ = 1.74 × 10^−5^						
		*E* = 0.170 kJ mol^−1^						
Ponceau S	Dubinin–Radushkevich	*q*_m_ = 2.20 × 10^6^ mg g^−1^	0.716	30469.54	78.06	2.12 × 10^15^	60.00	9390.10
		*K*_DR_ = 8.21 × 10^−5^						
		*E* = 0.078 kJ mol^−1^						
Trypan blue	Sips	*q*_m_ = 1.00 × 10^8^ mg g^−1^	0.877	13116.52	51.22	65361.51	41.52	7165.84
		*K*_s_ = 5.26 × 10^−3^ L mg^−1^						
		*n*_s_ = 3.787						
Ponceau S	Sips	*q*_m_ = 1.00 × 10^8^ mg g^−1^	0.718	30232.90	77.76	427.52	48.90	7966.36
		*K*_s_ = 2.88 × 10^−4^ L mg^−1^						
		*n*_s_ = 2.030						
Trypan blue	Redlich–Peterson	*K*_RP_ = 44.80 L g^−1^	0.674	34690.42	83.30	136.19	39.25	8543.69
		*a*_RP_ = 1.69 × 10^−12^						
		*β* = 1.00 × 10^−6^						
Ponceau S	Redlich–Peterson	*K*_RP_ = 43.90 L g^−1^	0.643	38287.80	87.51	139.91	36.61	8585.79
		*a*_RP_ = 8.34 × 10^−11^						
		*β* = 1.00 × 10^−6^						

For Trypan Blue, the adsorption capacity increased from 46.99 mg g^−1^ at 10 mg L^−1^ to 463.08 mg g^−1^ at 100 mg L^−1^. Similarly, the adsorption capacity of Ponceau S increased from 45.42 to 463.85 mg g^−1^ over the same initial concentration range. In addition, removal efficiencies for both dyes remained above 92% at 100 mg L^−1^. These results indicate that NiB exhibited substantial experimental dye-loading capacity within the investigated concentration range, although no distinct saturation plateau was observed.

The corrected equilibrium data were fitted using the non-linear forms of the Langmuir, Freundlich, Temkin, Dubinin–Radushkevich, Sips, and Redlich–Peterson models. The non-linear fitting profiles are presented in Fig. 5, and the corresponding model parameters and error-function values are summarized in Table 5. The non-linear approach was used as the primary fitting strategy because it preserves the original experimental *C*_e_–*q*_e_ relationship and avoids potential bias introduced by linear transformation.

**Fig. 5:**
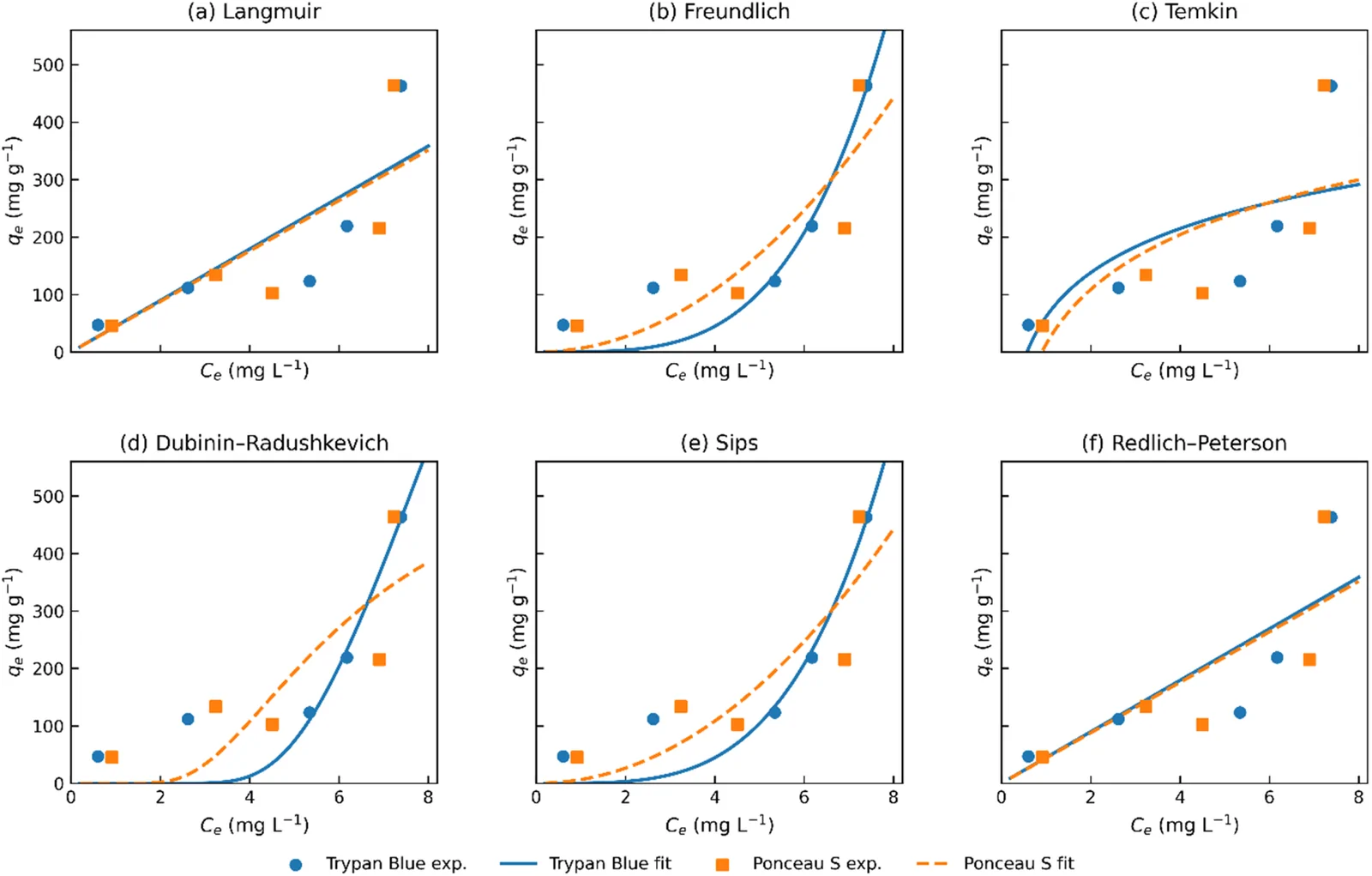
. Non-linear isotherm fitting of the equilibrium adsorption data for Trypan Blue and Ponceau S onto NiB: (a) Langmuir, (b) Freundlich, (c) Temkin, (d) Dubinin–Radushkevich, (e) Sips, and (f) Redlich–Peterson models. Symbols represent the experimental *C*_e_–*q*_e_ data, and solid lines represent the fitted non-linear model curves.

**Table 5:** Corrected experimental equilibrium data for Trypan Blue and Ponceau S adsorption onto NiB^a^

*C*_0_(mg L^−1^)	Trypan blue *C*_e_ (mg L^−1^)	Trypan blue removal (%)	Trypan blue *q*_e_ (mg g^−1^)	Ponceau S *C*_e_ (mg L^−1^)	Ponceau S removal (%)	Ponceau S *q*_e_ (mg g^−1^)
10	0.602	93.98	46.99	0.916	90.84	45.42
25	2.617	89.53	111.92	4.505	81.98	102.48
30	5.343	82.19	123.29	3.239	89.20	133.81
50	6.174	87.65	219.13	6.904	86.19	215.48
100	7.385	92.62	463.08	7.231	92.77	463.85

a *C*_0_ is the initial dye concentration, *C*_e_ is the equilibrium dye concentration, and *q*_e_ is the experimental equilibrium adsorption capacity. The *q*_e_ values were calculated using the corrected solution volume of 500 mL and an adsorbent mass of 0.1 g.

Model suitability was evaluated using *R*^2^, SSE, RMSE, *χ*^2^, ARE, and HYBRID values. Among the tested models, the Freundlich-type description provided the most appropriate overall representation of the equilibrium data, particularly when *R*^2^, SSE, and RMSE values were considered together. This result suggests that dye adsorption onto NiB was better described by a heterogeneous adsorption behavior than by an ideal monolayer saturation process within the investigated concentration range. This error-function-based interpretation is consistent with recent adsorption studies emphasizing that equilibrium model selection should be supported by model fit and residual error criteria rather than relying solely on linearized regression outputs.^6,14^

Although Langmuir- and Sips-type models can provide useful capacity-related estimates, the very high apparent *q*_max_ or *q*_m_ values obtained from these models indicate that the maximum adsorption capacity was not experimentally reached. Therefore, these capacity parameters were interpreted as apparent model-derived estimates rather than definitive maximum adsorption capacities. The highest experimental *q*_e_ values obtained at 100 mg L^−1^ were consequently considered more reliable indicators of adsorption performance than model-derived maximum capacity estimates.

Because Sips and Redlich–Peterson are three-parameter models, their fitting results were interpreted with caution. Although these models can describe heterogeneous adsorption behavior, the limited number of equilibrium points may increase the risk of overfitting. Therefore, the final isotherm interpretation was based on the combined evaluation of model parameters, error-function values, experimental *q*_e_ trends, and the absence of a clear saturation region.

### 3.5. Kinetic modeling and rate-controlling steps

Kinetic modeling was conducted to evaluate the adsorption rate and possible rate-controlling steps involved in the removal of Trypan Blue and Ponceau S by NiB. The *q*_*t*_–time profiles are presented in Fig. 6. For both dyes, *q*_*t*_ increased rapidly during the initial adsorption stage, reaching maximum or near-maximum values at approximately 60 min, followed by limited fluctuations at longer contact times. This kinetic pattern suggests a rapid initial uptake stage associated with the availability of accessible surface sites, followed by a slower stage related to adsorption–desorption equilibration, surface rearrangement, and diffusion-related resistance.

**Fig. 6:**
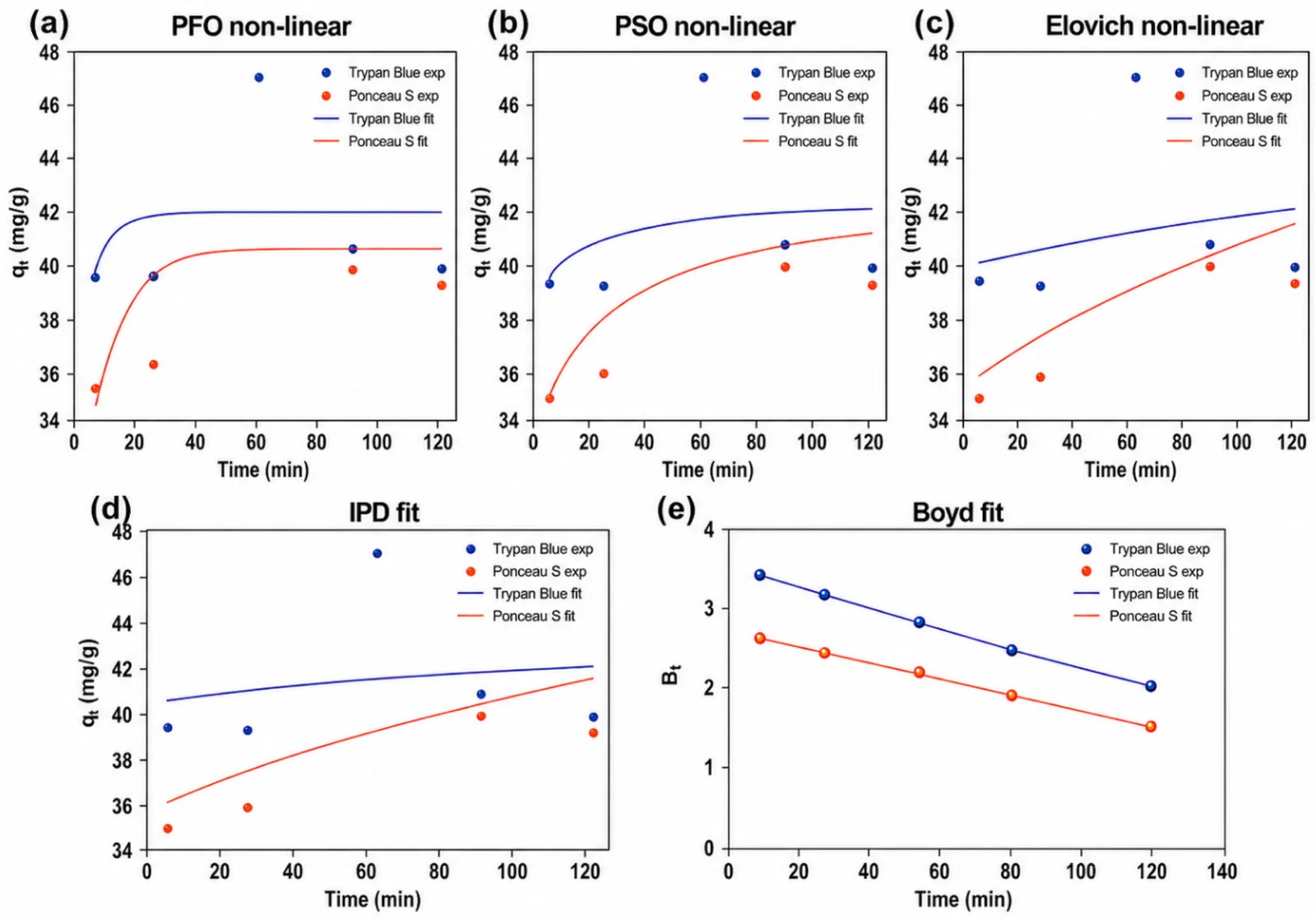
Non-linear kinetic model fitting and Boyd diffusion analysis for Trypan Blue and Ponceau S adsorption onto NiB: (a) pseudo-first-order model, (b) pseudo-second-order model, (c) Elovich model, (d) intraparticle diffusion model, and (e) Boyd plot. Symbols represent experimental data, and solid lines represent the fitted model curves.

The kinetic data were re-evaluated using the non-linear forms of the pseudo-first-order, pseudo-second-order, Elovich, and intraparticle diffusion models. Boyd analysis was also included as a diffusion diagnostic approach to assess the possible contribution of film diffusion and particle diffusion to the overall adsorption rate. The non-linear kinetic fitting profiles are presented in Fig. 6, and the corresponding kinetic model parameters and goodness-of-fit/error-function values are summarized in Table 6. Model suitability was evaluated using *R*^2^ together with SSE, RMSE, *χ*^2^, ARE, and HYBRID values. Therefore, kinetic model selection was based on the combined evaluation of regression quality and error-function criteria rather than on *R*^2^ alone. This approach provides a more reliable basis for kinetic interpretation because linear transformation of kinetic equations can modify the original error distribution and bias parameter estimation.^24,30,39,40^

**Table 6:** Non-linear kinetic model parameters and goodness-of-fit/error-function values for Trypan Blue and Ponceau S adsorption onto NiB^a^

Model	Parameter	Trypan blue	Ponceau S
Pseudo-first-order	*q*_e_,_cal_ (mg g^−1^)	41.832	40.654
	*k*_1_ (min^−1^)	0.1850	0.1231
	*R* ^2^	0.117	0.398
	SSE	35.015	37.487
	RMSE	2.646	2.738
	*χ* ^2^	0.838	0.935
	ARE (%)	4.917	5.653
	HYBRID	26.123	30.434
Pseudo-second-order	*q*_e_,_cal_ (mg g^−1^)	42.505	42.407
	*k*_2_ (g mg^−1^ min^−1^)	0.02119	0.00743
	*R* ^2^	0.113	0.437
	SSE	35.184	35.044
	RMSE	2.653	2.647
	*χ* ^2^	0.843	0.874
	ARE (%)	4.753	5.152
	HYBRID	26.118	27.305
Elovich	*α* (mg g^−1^ min^−1^)	2.415 × 10^19^	7.207 × 10^4^
	*β* (g mg^−1^)	1.1796	0.3588
	*R* ^2^	0.052	0.356
	SSE	37.604	40.103
	RMSE	2.742	2.832
	*χ* ^2^	0.906	1.007
	ARE (%)	5.024	5.371
	HYBRID	27.775	30.806
Intraparticle diffusion	*k*_id_ (mg g^−1^ min^−1/2^)	0.1545	0.7212
	*C*	40.120	33.766
	*R* ^2^	0.020	0.277
	SSE	38.863	45.015
	RMSE	2.788	3.000
	*χ* ^2^	0.940	1.142
	ARE (%)	5.148	5.765
	HYBRID	28.667	34.601
Boyd	Slope	−0.00024	0.00433
	Intercept	1.922	1.566
	*R* ^2^	0.0001	0.0205
	*q*-Based error functions	Not applicable	Not applicable

a The kinetic model parameters were obtained by direct non-linear fitting of the experimental *q*_*t*_–*t* data. Model suitability was evaluated using *R*^2^ together with SSE, RMSE, *χ*^2^, ARE, and HYBRID values. Boyd analysis was used to evaluate diffusion-related rate-control behavior; therefore, *q*_*t*_-based error functions were not applied to the Boyd model.

The non-linear, error-function-based evaluation indicated that the classical monotonic kinetic models did not fully capture the slight decrease or fluctuations in *q*_*t*_ values observed after 60 min. Although the non-linear pseudo-second-order model produced calculated equilibrium adsorption capacities close to the experimental values, the associated error-function values did not support an exclusive single-mechanism interpretation. Therefore, the adsorption kinetics should not be assigned solely to chemisorption control. The limited fitting performance of the Elovich model suggests that surface heterogeneity and site-energy distribution may contribute to the adsorption process, but these effects did not dominate the overall kinetic behavior. Similarly, the low fitting performance of the intraparticle diffusion model indicates that intraparticle diffusion was not the sole rate-controlling step. Boyd analysis further suggested that particle diffusion alone did not control the adsorption process. Overall, the combined evaluation of experimental and calculated *q*_*t*_ values, non-linear fitting profiles, and error-function values supports a multi-step adsorption process involving rapid surface attachment, external film diffusion, surface rearrangement, and limited intraparticle or interparticle diffusion rather than a single rate-controlling mechanism. Accordingly, the kinetic models were interpreted alongside non-linear regression, error-function analysis, pH-dependent adsorption behavior, FTIR evidence, and thermodynamic trends, thereby providing a more reliable basis for mechanistic evaluation.^30,41–43^

### 3.6. Regeneration and reuse performance

Regeneration and reuse experiments were performed to evaluate the practical reusability of NiB after adsorption of Trypan Blue and Ponceau S. Two regeneration methods were compared: chemical regeneration with HCl and thermal regeneration. The adsorbent was tested over nine consecutive adsorption–regeneration cycles, and the results are shown in Fig. 7. For HCl regeneration, Trypan Blue removal decreased sharply from 90.3% in the first cycle to 3.5% in the ninth cycle, while Ponceau S removal decreased from 84.0% to 8.7%. This pronounced loss of adsorption performance may be associated with partial alteration of acid-sensitive surface sites, incomplete dye desorption, changes in the surface protonation state, or loss of adsorption-active functionalities. Because post-regeneration structural characterization was not performed after each cycle, possible changes in the NiB framework were interpreted as a plausible explanation rather than as a confirmed structural transformation. In contrast, thermal regeneration maintained considerably higher reuse performance. After the ninth cycle, Trypan Blue removal remained at 72.5%, and Ponceau S removal remained at 68.0%. These values correspond to approximately 75% and 74% retention of the initial removal performance, respectively (Fig. 7). The better thermal regeneration performance indicates that thermal treatment was more effective than HCl treatment in restoring adsorption sites while preserving the functional integrity of the NiB surface. Recent adsorption studies have highlighted that adsorption performance should be evaluated not only by removal efficiency and adsorption capacity but also by regeneration behavior, post-use management, and applicability under continuous-flow or fixed-bed conditions.^5,6^ Previous studies have also shown that acid or chemical regeneration can cause active-site loss, incomplete desorption, or structural/surface changes in clay, metal oxide, carbon-based, and composite adsorbents, whereas properly controlled thermal regeneration can maintain reuse capacity over multiple cycles.^39,44–46^

**Fig. 7:**
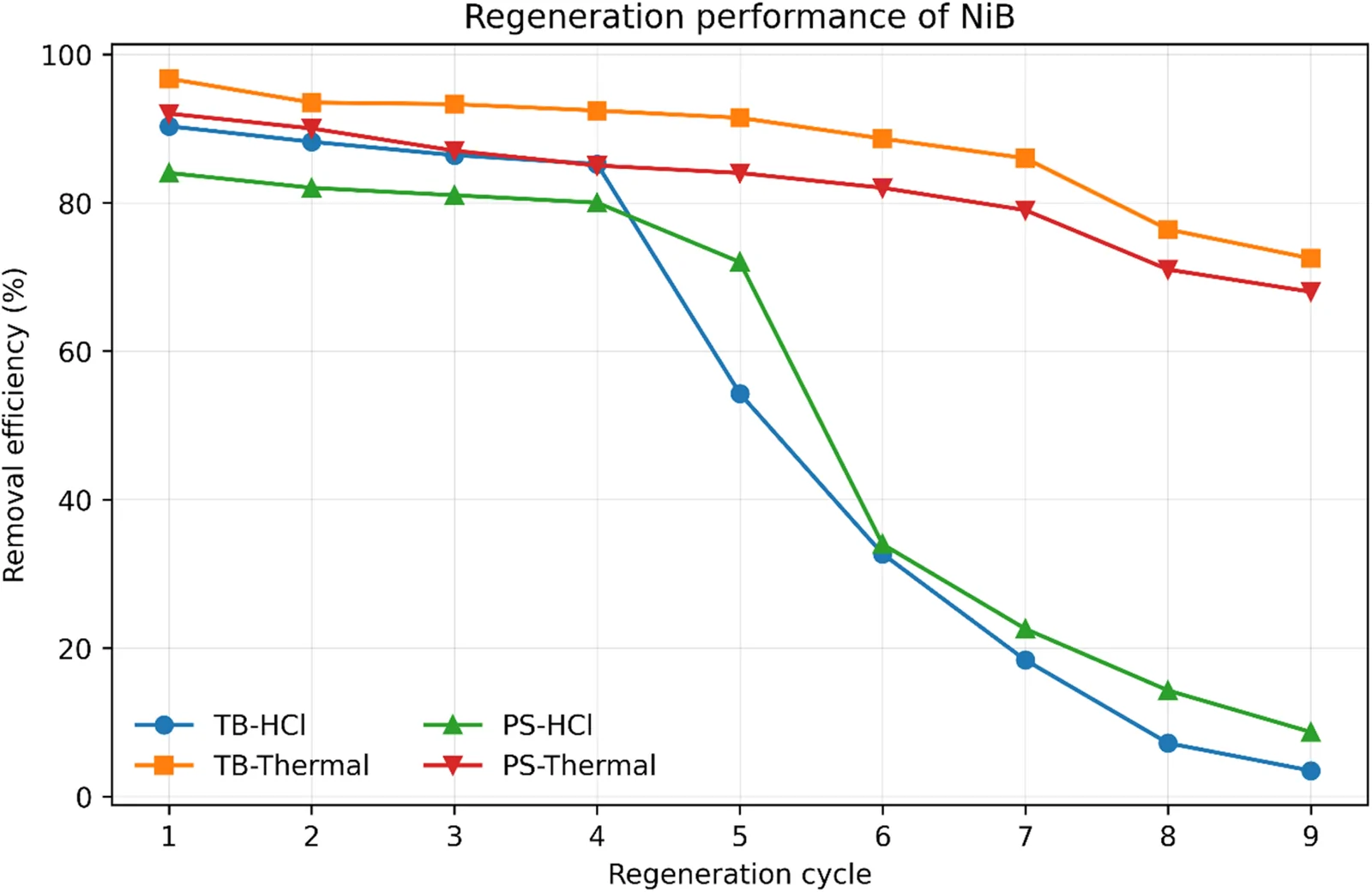
. Reuse performance of NiB for Trypan Blue and Ponceau S removal after HCl and thermal regeneration over nine consecutive adsorption–regeneration cycles.

### 3.7. Proposed adsorption mechanism

The adsorption of Trypan Blue and Ponceau S onto NiB appears to involve multiple complementary interactions rather than a single mechanism. The pH experiments showed that high removal efficiencies were obtained not only under acidic conditions but also under neutral and alkaline conditions (Fig. 3b). This behavior suggests that electrostatic attraction may contribute to the initial adsorption of anionic dye molecules, but it is unlikely to be the sole governing interaction. Because direct pH_zpc_ and zeta potential measurements were not performed, electrostatic attraction was interpreted as a plausible contributing mechanism based on the pH-dependent adsorption trend rather than as a directly confirmed surface-charge-driven process. FTIR spectral changes in the B–O, Ni–O, hydroxyl-related, and dye-associated regions further suggest that surface functional groups participate in dye retention (Fig. 1b). Kinetic modeling supported this interpretation. The better fit of the pseudo-second-order model relative to the pseudo-first-order and intraparticle diffusion models indicated that surface interactions played an important role, whereas the low intraparticle diffusion *R*^2^ values indicated that intra-pore diffusion alone was not rate-limiting (Fig. 6). However, pseudo-second-order fitting was not considered direct evidence of chemisorption. Instead, the kinetic results suggest the coexistence of external film diffusion, rapid surface binding, and limited diffusion-related steps. Thermodynamic results also supported the formation of stable dye–surface interactions at lower temperatures, as reflected by negative Δ*G*° and Δ*H*° values and decreasing removal efficiency with increasing temperature (Fig. 3e). Regeneration results provided additional evidence: thermal regeneration preserved adsorption performance better than HCl regeneration, suggesting that stable yet regenerable surface interactions were involved (Fig. 7). Taken together, the adsorption mechanism can be described as a multi-step process involving the transport of dye molecules from the solution to the NiB surface, rapid attachment to accessible Ni–O, B–O, and hydroxyl-related sites, and subsequent surface rearrangement or limited diffusion. Electrostatic attraction, hydrogen bonding, Lewis acid–base interactions, possible surface complexation, and weak physical interactions may all contribute to dye retention. Similar multi-interaction mechanisms have been reported for dye and pollutant adsorption on nickel-containing inorganic materials, metal oxide systems, and hydroxylated adsorbent surfaces, where surface charge, metal–oxygen centers, hydroxyl groups, and adsorbate functional groups act together.^18,40,47,48^

### 3.8. Comparison with related adsorbents and implications

Because studies specifically addressing Trypan Blue and Ponceau S removal by NiB are limited, the present results were evaluated within the broader context of borate-based and related inorganic adsorbents. Recent studies on borate materials such as SrAl_2_B_2_O_7_, Na_2_Al_2_B_2_O_7_, Ba_2_Mg(BO_3_)_2_, and Sr_2_Mg(BO_3_)_2_ alkaline-earth metal borates such as Ba_2_Mg(BO_3_)_2_ and Sr_2_Mg(BO_3_)_2_ have shown that metal borate structures can function as promising adsorbent platforms for anionic dye removal.^20–22,49^ To position the present NiB adsorbent within this broader literature, its adsorption performance was compared with selected adsorbents reported for anionic dye removal, with particular attention to adsorption capacity, contact time, adsorbent dose, operating pH, and regeneration behavior (Table 7).

**Table 7:** Comparison of NiB with selected adsorbents reported for anionic dye removal

Dye	Adsorbent	Time (min)	Dose (mg L^−1^)	Initial dye dose (mg L^−1^)	Removal yield (%)	*q*_e_ (mg g^−1^)	pH	*T* (°C)	References
Trypan blue	Sr_2_Ni(BO_3_)_2_	60	200	100	>92	463.08	7.37	25	This study
Ponceau S		60	200	100	>92	463.85	7.52	25	This study
Reactive yellow 145	Catechin–PG–Fe complex	200	600	260	N/A	345	1	N/A	Hatimi *et al.* (2023)^50^
Reactive yellow 145	nZVI/(Fe–Mn) nanocomposite	40	1000	300	86	256	6	30	Lam *et al.* (2022)^51^
Reactive yellow 145	Chitosan functionalized by talc-Cloisite 30B	240	570	40	98.4	76.9	4	N/A	Kandil and Ali (2023)^52^
Reactive yellow 145	MgO nanoparticles	75	1000	100	65.5	59	3	25	Munir *et al.* (2023)^53^
Reactive yellow 145	Hydroxide-rich polypropylene hybrid microparticles	60	500	10	94	39.6	7	25	Bakry and Elmesallamy (2025)^54^
Reactive yellow 145	Multibranched α-Fe_2_O_3_ powder	60	500	20	50	32	3.5	25	Ramírez-Gómez *et al.* (2024)^55^
Reactive yellow 145	Sr_2_Mg(BO_3_)_2_	30	600	10	85.6	22.03	5.42	25	Esmeray *et al.* (2026)^20^
Reactive yellow 145	Treated watermelon seeds	180	1000	20	84	17.5	3	25	Benkaddour *et al.* (2018)^56^
Reactive yellow 145	Groundnut shell biochar	360	1000	15	62	7.3	2	35	Krishnasamy *et al.* (2022)^57^

In the present study, the high removal efficiencies obtained for both Trypan Blue and Ponceau S, the high corrected q_e_ values at increasing initial dye concentrations, and the relatively stable performance after thermal regeneration indicate that NiB is a promising reusable inorganic adsorbent for sulfonated anionic dyes. The comparison in Table 7 shows that several highly engineered or chemically modified adsorbents reported for the removal of Reactive Yellow 145 exhibit high adsorption capacities; however, these systems often require strongly acidic conditions, longer contact times, higher adsorbent dosages, or more complex synthesis routes. For example, high-capacity systems such as the catechin–propylene glycol–Fe complex and surfactant-modified activated carbon require highly acidic conditions, whereas NiB achieved high experimental *q*_e_ values for both Trypan Blue and Ponceau S under near-neutral conditions. Although the present dyes differ from Reactive Yellow 145, they are all sulfonated anionic dyes, and this comparison provides a useful contextual benchmark for evaluating NiB performance. The results suggest that NiB may offer a favorable balance between adsorption capacity, moderate contact time, near-neutral operation, and regeneration potential. However, the isotherm results also demonstrate that the current concentration range did not produce a clear saturation plateau. Therefore, Langmuir-type *q*_max_ values were evaluated as model-derived parameters rather than definitive adsorption capacity limits. For this reason, the adsorption capacities reported in Table 7 of the present study represent the highest experimental *q*_e_ values obtained at 100 mg L^−1^, not the definitive maximum adsorption capacities. Further investigation at higher initial dye concentrations is required to more accurately define the saturation region and verify the maximum loading potential of NiB. In addition, direct comparisons with adsorbents reported in the literature should be interpreted with caution because the reported studies differ in dye structure, initial concentration, solution chemistry, adsorbent dose, contact time, pH, temperature, and regeneration strategy. Although the present findings demonstrate the high adsorption performance and reusability potential of NiB, further analyses are required to confirm its practical applicability under real treatment conditions. In particular, BET surface area and pore-size distribution analyses are needed to relate adsorption performance to textural properties, while XPS analysis would provide more direct evidence for surface chemical interactions between dye molecules and NiB. In addition, Ni and B leaching tests should be conducted to evaluate the environmental stability and safety of the adsorbent during repeated use. Future studies should also examine NiB performance in real textile wastewater, multi-dye systems, competing-ion matrices, and continuous-flow column operation to better assess the feasibility of scale-up. Overall, these findings support the further development of NiB as a reusable inorganic adsorbent for the removal of resistant anionic dyes.

## 4. Conclusion

This study demonstrated the applicability of synthesized Sr_2_Ni(BO_3_)_2_, abbreviated as NiB, as a reusable inorganic adsorbent for the removal of Trypan Blue and Ponceau S from aqueous solutions. High removal efficiencies were achieved for both dyes under near-neutral conditions, at 250 rpm and 25 °C, with rapid adsorption occurring within the first 60 min. Corrected equilibrium calculations indicated high experimental adsorption capacities of 463.08 mg g^−1^ for Trypan Blue and 463.85 mg g^−1^ for Ponceau S at an initial dye concentration of 100 mg L^−1^, with removal efficiencies remaining above 92%. Non-linear isotherm evaluation confirmed that NiB exhibited high dye-loading capacity within the tested concentration range. However, because the equilibrium data did not reach a clear saturation plateau, model-derived *q*_max_ values were interpreted as apparent estimates rather than definitive maximum adsorption capacities. The absence of a clear saturation plateau indicates that further high-concentration isotherm experiments are needed to more accurately define the saturation region and maximum adsorption capacity of NiB.

The non-linear kinetic evaluation and error-function analysis supported a multi-step adsorption mechanism involving rapid surface attachment, surface interactions, surface rearrangement, and mass-transfer contributions rather than a single rate-controlling process. pH-dependent adsorption behavior, FTIR spectral changes, kinetic modeling, and thermodynamic parameters suggested the involvement of electrostatic attraction, hydrogen bonding, Lewis acid–base interactions, possible surface complexation, and diffusion-related effects. Thermodynamic results indicated spontaneous and exothermic adsorption. Thermal regeneration was more effective than HCl regeneration, maintaining removal efficiencies of 72.5% for Trypan Blue and 68.0% for Ponceau S after nine cycles. Together, the adsorption, non-linear modeling, thermodynamic, and regeneration results indicate that NiB has potential as a reusable inorganic adsorbent; however, its practical applicability should be further assessed beyond batch conditions. These findings support further evaluation of NiB for anionic dye removal, particularly under real wastewater conditions, in competitive-ion environments, under continuous-flow or fixed-bed operation, for post-use management, for adsorbent stability, and for Ni/B leaching assessment.

## Author contributions

Ayhan Kocaman: conceptualization, methodology, supervision, validation, writing—review and editing, project administration, corresponding author. Ertuğrul Esmeray: conceptualization, methodology, investigation, data curation, formal analysis, visualization, writing—original draft, writing—review and editing. Hakan Çelebi: methodology, investigation, formal analysis, validation, writing—review and editing. Mustafa Karataş: methodology, investigation, data curation, formal analysis, writing—review and editing. İlhan Pekgözlü: conceptualization, methodology, supervision, validation, writing—review and editing. All authors read and approved of the final manuscript.

## Conflicts of interest

The authors declare that they have no competing interests.

## Data Availability

The data supporting the findings of this study are included within the article, including the experimental data presented in the figures and tables. No additional datasets have been deposited in an external repository.

## References

[cit1] Fahad A., Darwish M., Ethaib S., Aris A., Ali I. M., Ali N., Mustafa S. (2026). Innovative approaches to textile wastewater treatment: State-of-the-art technologies and future horizons.. Sci. Afr..

[cit2] Beulah S. S., Muthukumaran K. (2020). Methodologies of removal of dyes from wastewater: A review. Int. Res. J. Pure Appl. Chem..

[cit3] Bora N., Joshi D. P., Sharma R. (2026). Contamination toxicity and remediation of stable dyes and oils in aquatic wastewater systems. Discover Environ..

[cit4] Danu B. Y., Bandoh C. K., Adusei J. K., Haruna M., Kangmennaa A., Yeboah P., Ampong F. K., Agorku E. S. (2026). Carbon-based materials for the removal of organic dyes from wastewater. Discover Nano.

[cit5] Hussein E. B., Rasheed F. A. (2025). Innovative use of recycled aluminum adsorbent for methylene blue adsorption and post-application for soil stabilization. J. Contam. Hydrol..

[cit6] Hussein E. B., Rasheed F. A. (2025). Sustainable removal of methylene blue from wastewater using silica sand coated with Al2O3 nanoparticles: a comparative study of batch and fixed bed column reactors. RSC Adv..

[cit7] Adeyi A. A., Fasina F. D., Giwa A. (2018). Kinetics and Isotherm Studies of liquid phase adsorption of cationic dye onto chitosan synthesized from crab shells. Int. J. Eng. Res. Afr..

[cit8] Al-Wasidi A. S., Abdelrahman E. A., Rehman K., Saad F. A., Munshi A. M. (2024). Efficient removal of crystal violet and acid red 88 dyes from aqueous environments using easily synthesized copper ferrite nanoparticles. Sci. Rep..

[cit9] Jiang B., Feng S. H., Zhang J. P. (2022). The adsorption and regeneration of magnetic modified bentonite composite for dye wastewater. Key Eng. Mater..

[cit10] Le Godec Y., Courac A. (2021). In situ high-pressure synthesis of new outstanding light-element materials under industrial P-T range. Materials.

[cit11] Sari F. P., Yanto D. H. Y., Pari G. (2019). Activated carbon derived from OPEFB by one step steam activation and its application for dye adsorption: kinetics and isothermal studies. Reaktor.

[cit12] Soran M.-L., Bocşa M., Pintea S., Stegarescu A., Lung I., Opriş O. (2023). Commercially biochar applied for tartrazine removal from aqueous solutions. Appl. Sci..

[cit13] Hussein E. B., Rasheed F. A., Mohammed A. S., Kayani K. F. (2025). Emerging nanotechnology approaches for sustainable water treatment and heavy metals removal: a comprehensive review. RSC Adv..

[cit14] Hussein E. B., Rasheed F. A. (2025). Enhanced amoxicillin adsorption from wastewater using KOH-modified Iraqi Silica Sand: Characterization, kinetics, and thermodynamics. Int. J. Environ. Res..

[cit15] Kousar S., Abdel R. M., Zaki M. E., Khairy M., El-Aassar M. R., Alanazi A. K., Shakir I., Mubeen S., Aadil M. (2025). Towards advanced remediation: CuS/CdS Z-scheme heterojunction with accelerated charge-transfer for efficient photocatalytic degradation of trypan blue. RSC Adv..

[cit16] Marshall T., Lamont K., Marangoni A. G., Lim L.-T., Wang X., Pensini E. (2021). Trypan blue removal from water with zein sorbents and laccase. SN Appl. Sci..

[cit17] Bhagyaraj S., Al-Ghouti M. A., Kasak P., Krupa I. (2021). An updated review on boron removal from water through adsorption processes. Emergent Mater..

[cit18] Patil Y., Attarde S., Dhake R., Fegade U., Alaghaz A. N. M. (2023). Adsorption of Congo red dye using metal oxide nano-adsorbents: Past, present, and future perspective. Int. J. Chem. Kinet..

[cit19] Wang J., Zhang Y., Liu F., Liu Y., Wang L., Gao G. (2023). Preparation of a multifunctional and multipurpose chitosan/cyclodextrin/MIL-68 (Al) foam column and examining its adsorption properties for anionic and cationic dyes and sulfonamides. ACS Omega.

[cit20] Esmeray E., Özkök E., Çelebi H., Karataş M., Pekgözlü İ. (2026). Decolorization of Anionic Dye RY-145 via Combustion-Synthesized Sr2Mg (BO3) 2: Comparative Linear and Non-linear Isotherm and Kinetic Modelling. J. Indian Chem. Soc..

[cit21] Esmeray E., Özkök E., Çelebi H., Karataş M., Pekgözlü İ. (2025). Synthesis and Application of Boron-Based Sr2Mg (BO3) 2 for the Efficient Adsorptive Removal of Reactive Red 195 from Aqueous Solutions. ChemistrySelect.

[cit22] Şentürk İ., Pekgözlü İ. (2025). Preparation, characterization and adsorption properties of Ba2Mg (BO3) 2 adsorbent for anionic dye Acid Violet. J. Indian Chem. Soc..

[cit23] Nistor M.-A., Muntean S. G., Ianoş R., Racoviceanu R., Ianaşi C., Cseh L. (2021). Adsorption of anionic dyes from wastewater onto magnetic nanocomposite powders synthesized by combustion method. Appl. Sci..

[cit24] Abdelkhalek A., El-Latif M. A., Ibrahim H., Hamad H., Showman M. (2022). Controlled synthesis of graphene oxide/silica hybrid nanocomposites for removal of aromatic pollutants in water. Sci. Rep..

[cit25] Balasooriya I. L., Chen J., Korale Gedara S. M., Han Y., Wickramaratne M. N. (2022). Applications of nano hydroxyapatite as adsorbents: a review. Nanomaterials.

[cit26] Bouazizi N., Vieillard J., Samir B., Le Derf F. (2022). Advances in amine-surface functionalization of inorganic adsorbents for water treatment and antimicrobial activities: a review. Polymers.

[cit27] Liu X., Pang H., Liu X., Li Q., Zhang N., Mao L., Qiu M., Hu B., Yang H., Wang X. (2021). Orderly porous covalent organic frameworks-based materials: superior adsorbents for pollutants removal from aqueous solutions. Innovation.

[cit28] Yap P. L., Nine M. J., Hassan K., Tung T. T., Tran D. N., Losic D. (2021). Graphene-based sorbents for multipollutants removal in water: a review of recent progress. Adv. Funct. Mater..

[cit29] Zolezzi C., Ihle C. F., Angulo C., Palma P., Palza H. (2018). Effect of the oxidation degree of graphene oxides on their adsorption, flocculation, and antibacterial behavior. Ind. Eng. Chem. Res..

[cit30] Gong Y., Qin C., He W., Qiao Z., Zhang G., Chen R., Gao Y., Xiao L., Jia S. (2017). Solar light assisted green synthesis of photoreduced graphene oxide for the high-efficiency adsorption of anionic dyes. RSC Adv..

[cit31] Castro L. E. Nd, Battocchio D. A. J., Ribeiro L. F., Colpini L. M. S. (2023). Development of adsorbent materials using residue from coffee industry and application in food dye adsorption processes. Braz. Arch. Biol. Technol..

[cit32] Magdy A., Zaki M. E., Mostafa M. R., Mohamed G. G., Al-Hussain S. A., Fouad O. A. (2025). Integrated kinetic, thermodynamic, and statistical investigation of aniline blue dye removal using magnesium silicate nanoparticles. Sci. Rep..

[cit33] Abidi N., Duplay J., Jada A., Errais E., Ghazi M., Semhi K., Trabelsi-Ayadi M. (2019). Removal of anionic dye from textile industries’ effluents by using Tunisian clays as adsorbents. Zeta potential and streaming-induced potential measurements. C. R. Chim..

[cit34] Bapat S., Jaspal D., Malviya A. (2021). Efficacy of parthenium hysterophorus waste biomass compared with activated charcoal for the removal of CI Reactive Red 239 textile dye from wastewater. Color. Technol..

[cit35] Ibrahim A. J., Dwesh H. A. W., Al-Sawad A. R. (2023). Adsorption of methylene blue dye onto bentonite clay: Characterization, adsorption isotherms, and thermodynamics study by using UV-Vis technique. Anal. Methods Environ. Chem. J..

[cit36] Lawtae P., Tangsathitkulchai C. (2021). The use of high surface area mesoporous-activated carbon from longan seed biomass for increasing capacity and kinetics of methylene blue adsorption from aqueous solution. Molecules.

[cit37] Raji Y., Nadi A., Rouway M., Jamoudi Sbai S., Yassine W., Elmahbouby A., Cherkaoui O., Zyade S. (2022). Efficient adsorption of methyl orange on nanoporous carbon from agricultural wastes: characterization, kinetics, thermodynamics, regeneration and adsorption mechanism. J. Compos. Sci..

[cit38] Sabzi M., Shafagh N., Mohammadi M. (2019). Assembly of gelatin biopolymer to fibrous sepiolite clay for efficient dye removal from wastewater. J. Appl. Polym. Sci..

[cit39] Momina, Shahadat M., Isamil S. (2018). Regeneration performance of clay-based adsorbents for the removal of industrial dyes: a review. RSC Adv..

[cit40] Ike J. I., Babayemi A. K., Egbosiuba T. C., Jin C. G., Mustapha S., Yusuff A. S., Yadav V. K., Igwegbe C. A. (2024). Treated kaolin clay incorporated with nickel nanoparticles for enhanced removal of crystal violet and methyl orange from textile wastewater. ACS Appl. Eng. Mater..

[cit41] Ghosh P., Chelli V. R., Giri A. S., Golder A. K. (2018). Steroid glycosides as potential analytes for Cu-doping on TiO2 for photocatalytic water treatment. Environ. Prog. Sustainable Energy.

[cit42] Showman M., Omara R., El-Ashtoukhy E. Z., Farag H., El-Latif M. A. (2024). Formulation of silver phosphate/graphene/silica nanocomposite for enhancing the photocatalytic degradation of trypan blue dye in aqueous solution. Sci. Rep..

[cit43] Wang C., Yin J., Wang R., Jiao T., Huang H., Zhou J., Zhang L., Peng Q. (2019). Facile preparation of self-assembled polydopamine-modified electrospun fibers for highly effective removal of organic dyes. Nanomaterials.

[cit44] Fdez-Sanromán A., Pazos M., Rosales E., Sanromán M. A. (2020). Unravelling the environmental application of biochar as low-cost biosorbent: a review. Appl. Sci..

[cit45] Gao S., Wang D., Huang Z., Su C., Chen M., Lin X. (2022). Recyclable NiO/sepiolite as adsorbent to remove organic dye and its regeneration. Sci. Rep..

[cit46] Reynel-Ávila H., Camacho-Aguilar K., Bonilla-Petriciolet A., Mendoza-Castillo D., González-Ponce H., Trejo-Valencia R. (2021). Engineered magnetic carbon-based adsorbents for the removal of water priority pollutants: an overview. Adsorpt. Sci. Technol..

[cit47] Marey A., Adel M., El Naggar A. M., El-Zahhar A. A., Taha M. H. (2023). Nickel-hydroxide-encapsulated polyacrylamide as a novel adsorptive composite for the capture of methylene blue from wastewater. Dalton Trans..

[cit48] Umeh C. T., Nduka J. K., Akpomie K. G., Ighalo J. O., Mogale R. (2025). Adsorptive effect of corn silk-loaded nickel oxide and copper oxide nanoparticles for elimination of ciprofloxacin from wastewater. ACS Omega.

[cit49] Bayram O., Moral E., Pekgözlü İ., Göde F. (2024). Technology: Use of Sr2Mg (BO3) 2 in Congo red removal from aqueous solutions: Synthesis, adsorption, isotherm, kinetic study. J. Dispersion Sci. Technol..

[cit50] Hatimi B., Loudiki A., Mouldar J., Hafdi H., Joudi M., Bensemlali M., Aarfane A., Nasrellah H., El Mhammedi M. A., Bakasse E. M. (2023). Physicochemical and statistical modeling of reactive Yellow 145 enhanced adsorption onto pyrrhotite ash-based novel (Catechin-PG-Fe)-complex. Mater. Sci. Energy Technol..

[cit51] Lam P. V., Duong N. B., Bich P. T. N., Trang Q. T. T. (2022). Adsorption removal of Reactive Yellow 145 dye from aqueous solution using novel nZVI/(Fe–Mn) binary oxide/bentonite nanocomposite. Desalin. Water Treat..

[cit52] Kandil H., Ali H. (2023). Simultaneous removal of cationic crystal violet and anionic reactive yellow dyes using eco-friendly chitosan functionalized by talc and Cloisite 30B. J. Polym. Environ..

[cit53] Munir R., Ali K., Naqvi S. A. Z., Maqsood M. A., Bashir M. Z., Noreen S. (2023). Biosynthesis of Leucaena leucocephala leaf mediated ZnO, CuO, MnO_2_, and MgO based nano-adsorbents for Reactive Golden Yellow-145 (RY-145) and Direct Red-31 (DR-31) dye removal from textile wastewater to reuse in agricultural purpose. Sep. Purif. Technol..

[cit54] Bakry A., Elmesallamy S. M. (2025). Hydroxide-rich polypropylene hybrid microparticles for the removal of reactive yellow 145 from aqueous solutions: equilibrium, kinetic and thermodynamic studies. BMC Chem..

[cit55] Ramírez-Gómez W. M., Garzón-Cucaita V., Carriazo J. G. (2024). Synthesis of iron oxide microparticles with fern leaf morphology: Assessment of the RY-145 azo-dye adsorption. Surf. Interfaces.

[cit56] Benkaddour S., Slimani R., Hiyane H., El Ouahabi I., Hachoumi I., El Antri S., Lazar S. (2018). Removal of reactive yellow 145 by adsorption onto treated watermelon seeds: kinetic and isotherm studies. Sustainable Chem. Pharm..

[cit57] Krishnasamy S., SaiAtchyuth B. A., Ravindiran G., Subramaniyan B., Ramalingam M., Sai Vamsi J. U. B., Ramesh B., Ramesh B., Razack N. A. (2022). Effective removal of reactive yellow 145 (RY145) using biochar derived from groundnut shell. Adv. Mater. Sci. Eng..

